# Arabinoxylan-Oligosaccharides Act as Damage Associated Molecular Patterns in Plants Regulating Disease Resistance

**DOI:** 10.3389/fpls.2020.01210

**Published:** 2020-08-07

**Authors:** Hugo Mélida, Laura Bacete, Colin Ruprecht, Diego Rebaque, Irene del Hierro, Gemma López, Frédéric Brunner, Fabian Pfrengle, Antonio Molina

**Affiliations:** ^1^ Centro de Biotecnología y Genómica de Plantas, Universidad Politécnica de Madrid (UPM)—Instituto Nacional de Investigación y Tecnología Agraria y Alimentaria (INIA), Pozuelo de Alarcón (Madrid), Spain; ^2^ Departamento de Biotecnología-Biología Vegetal, Escuela Técnica Superior de Ingeniería Agronómica, Alimentaría y de Biosistemas, UPM, Madrid, Spain; ^3^ Department of Biomolecular Systems, Max Planck Institute of Colloids and Interfaces, Potsdam, Germany; ^4^ PlantResponse Biotech S.L., Campus de Montegancedo UPM, Pozuelo de Alarcón (Madrid), Spain

**Keywords:** arabinoxylan, cell wall, damage-associated molecular pattern (DAMP), plant immunity, pattern triggered immunity

## Abstract

Immune responses in plants can be triggered by damage/microbe-associated molecular patterns (DAMPs/MAMPs) upon recognition by plant pattern recognition receptors (PRRs). DAMPs are signaling molecules synthesized by plants or released from host cellular structures (e.g., plant cell walls) upon pathogen infection or wounding. Despite the hypothesized important role of plant cell wall-derived DAMPs in plant-pathogen interactions, a very limited number of these DAMPs are well characterized. Recent work demonstrated that pectin-enriched cell wall fractions extracted from the cell wall mutant impaired in *Arabidopsis Response Regulator 6* (*arr6*), that showed altered disease resistance to several pathogens, triggered more intense immune responses than those activated by similar cell wall fractions from wild-type plants. It was hypothesized that *arr6* cell wall fractions could be differentially enriched in DAMPs. In this work, we describe the characterization of the previous immune-active fractions of *arr6* showing the highest triggering capacities upon further fractionation by chromatographic means. These analyses pointed to a role of pentose-based oligosaccharides triggering plant immune responses. The characterization of several pentose-based oligosaccharide structures revealed that β-1,4-xylooligosaccharides of specific degrees of polymerization and carrying arabinose decorations are sensed as DAMPs by plants. Moreover, the pentasaccharide 3^3^-α-L-arabinofuranosyl-xylotetraose (XA3XX) was found as a highly active DAMP structure triggering strong immune responses in *Arabidopsis thaliana* and enhancing crop disease resistance.

## Introduction

Plants are sessile organisms that need to develop robust disease resistance mechanisms to efficiently defend from pathogens and pests. Activation of plant defense responses requires the perception of molecules from the pathogen (“non-self” signals) and from the plant (“damaged-self” signals) that trigger specific resistance responses through diverse molecular monitoring systems (Atkinson and Urwin, 2012). Among these monitoring mechanisms are pattern- and effector-triggered immunity (PTI and ETI) (Dodds and Rathjen, 2010). PTI is based in the recognition by pattern recognition receptors (PRRs) of microbe/pathogen-associated molecular patterns (MAMPs/PAMPs) from microorganisms or of plant-derived damage-associated molecular patterns (DAMPs) (Boller and Felix, 2009). MAMPs and DAMPs structures, with different biochemical composition (e.g., proteins, carbohydrates, lipids, and nucleic acids) have been identified, thus reflecting the diversity of immunogenic structures recognized by plants (Boutrot and Zipfel, 2017). In comparison with the high number of MAMPs characterized so far, much less DAMPs derived from plants have been identified to date (Choi and Klessig, 2016; Duran-Flores and Heil, 2016; Bacete et al., 2018; De Lorenzo et al., 2018; Li et al., 2020).

The plant cell wall is a dynamic and highly regulated structure mainly consisting of carbohydrate-based polymers, essential for growth, and development (Srivastava et al., 2017). Cellulose is the main load-bearing component in all plant cell walls, whereas different types of hemicelluloses and pectins are found in different plant phylogenetic groups (Carpita and Gibeaut, 1993; Carpita and McCann, 2000). Xylans are a diverse group of hemicelluloses with the common feature of a backbone of β-1,4-linked xylose residues (Scheller and Ulvskov, 2010). Monocot xylans usually contain many arabinose residues attached to the backbone and are known as arabinoxylans (AXs). Arabinofuranose substitutions are, in principle, less frequent in dicot xylans, but exceptions are found (Darvill et al., 1980; Fischer et al., 2004; Naran et al., 2008). Instead, dicot xylans are more commonly substituted with α-1,2-linked glucuronosyl and 4-*O*-methyl glucuronosyl residues known as glucuronoxylans which are the dominating non-cellulosic polysaccharides in the secondary walls of dicots. This variability in the fine structure of wall polymers exists not only among phylogenetic groups of plants, but also even between different tissues of a given plant. Cell wall heterogeneity may have had an evolutionary impact in the diversity of mechanisms that pathogens have evolved to breach plant cell walls, including the secretion of numerous cell wall-degrading enzymes (CWDE), such as cellulases, polygalacturonases, or xylanases (Annis and Goodwin, 1997). The functional integrity of cell walls is controlled by cell wall integrity monitoring systems (Bacete et al., 2018; Vaahtera et al., 2019). These systems trigger countervailing responses to cell wall restructuring which occurs upon pathogen infection, abiotic stress, and cell expansion during growth and development. The plant cell wall integrity pathway is strongly involved in the regulation of growth, immune responses and resource allocation between development and immunity (Hamann et al., 2009; Wolf et al., 2012; Engelsdorf et al., 2018). Alterations in cell wall composition or integrity by genetic or chemical means have a significant impact on plant resistance to different pathogens and/or abiotic stresses, since they typically lead to the activation of defensive signaling pathways, some of which are regulated by hormones (Miedes et al., 2014; Mélida et al., 2015; Nafisi et al., 2015; Houston et al., 2016; Bacete et al., 2020). For example, enhanced resistance to pathogens has been observed in *Arabidopsis thaliana* (Arabidopsis) mutants defective in specific cellulose synthases, enzymes involved in xylan decoration and in lignin biosynthesis (Ellis et al., 2002; Hernández-Blanco et al., 2007; Delgado-Cerezo et al., 2012; Xu et al., 2014; Escudero et al., 2017; Gallego-Giraldo et al., 2020; Molina et al., 2020).

Given both the complexity of the plant cell wall and the fact that many pathogens secrete a wide range of CWDE, it would be expected that the breakdown products of cell wall polymers could act as DAMPs that regulate immune responses. Confirming this hypothesis, pectic oligogalacturonides (OGs) were first cell wall DAMPs to be characterized (Nothnagel et al., 1983). OGs are derived from homogalacturonan, the main component of pectins, as a result of the activity of CWDE released by the pathogens during the colonization process (Ridley et al., 2001; Benedetti et al., 2015; Voxeur et al., 2019). Also, the overexpression or inactivation of genes encoding enzymes involved in the control of pectin structure [e.g., pectin methyl esterases (PME) and PME inhibitors], results in the modification of the degree of OGs release upon infection, and alterations of disease resistance phenotypes (Ferrari et al., 2008; Raiola et al., 2011; Lionetti et al., 2017; Benedetti et al., 2018; De Lorenzo et al., 2019). Another group of cell wall-derived carbohydrates recently characterized as DAMPs in Arabidopsis are cellulose-derived oligomers (β-1,4-glucans), which trigger signaling cascades sharing many similarities with the responses activated by OGs (Aziz et al., 2007; Souza et al., 2017; Johnson et al., 2018; Locci et al., 2019). With around 20 different monosaccharide moieties building the polysaccharides of the plant cell wall, other carbohydrate-based cell wall molecules in addition to OGs and cello-oligosaccharides should have been selected by plants as DAMPs. In line with this hypothesis, recent works have also nominated xyloglucan and mannan cell wall-derived oligosaccharides as plant DAMPs (Claverie et al., 2018; Zang et al., 2019), and β-1,3-glucan oligosaccharides present in plant callose but also in fungal cell walls, as dual DAMPs/MAMPs (Mélida et al., 2018).

Thus, growing evidences have awarded the cell wall with prominent novel roles in plant immunity. In this line, we have recently proposed a novel link between the cytokinin signaling pathway, cell wall composition control, and disease resistance responses through Arabidopsis Response Regulator 6 (ARR6) protein (Bacete et al., 2020). Cytokinins have emerged as an important hub integrating defense responses mediated by other hormones, and have been shown to regulate the activation of immune responses (Choi et al., 2011). In Arabidopsis, cytokinins are perceived by Arabidopsis Histidine Kinase receptors, that are two-component system proteins which initiate a downstream phosphotransfer cascade that leads to the phosphorylation of Arabidopsis Response Regulator (ARR) proteins (To et al., 2007). We showed a novel function for ARR6, as impairment of *ARR6* gene affect plant cell wall composition, which impact plant-pathogen interactions, and might lead to the accumulation of differential or increased levels of DAMPs in *arr6* in comparison to wild-type plants that would favor a “defense-ready” state instead of a resting one. Remarkably, pectin-enriched cell wall fractions extracted from *arr6* cell walls triggered, when applied to wild-type Arabidopsis plants, more intense immune responses than those activated by similar wall fractions from wild-type plants, suggesting that *arr6* pectin fraction is enriched in wall-derived DAMPs. In an effort toward a better understanding of plant mechanisms involved in cell wall-mediated immunity, we have further purified *arr6* pectin fraction. Results from such purifications suggested that pentose-based oligosaccharides co-extracted with pectins (using calcium chelators) could play a role as plant DAMPs. Afterwards, we purified several pentose-based oligosaccharides, generated by enzymatic digestion from a natural material source rich in that type of hemicelluloses, that were biochemically analyzed and tested for their capacity to induce PTI hallmarks (Boller and Felix, 2009; Boudsocq et al., 2010; Ranf et al., 2011). Using this strategy, we identified AX-oligosaccharides as a novel group of DAMPs active on plants and characterized 3^3^-α-L-arabinofuranosyl-xylotetraose (XA3XX) as a highly active structure triggering strong immune responses in Arabidopsis and enhancing crop disease resistance.

## Results

### Low Molecular Weight Pectic Fractions From Arabidopsis Enriched in Oligopentoses Contain Active Plant DAMPs

**Figure 1:**
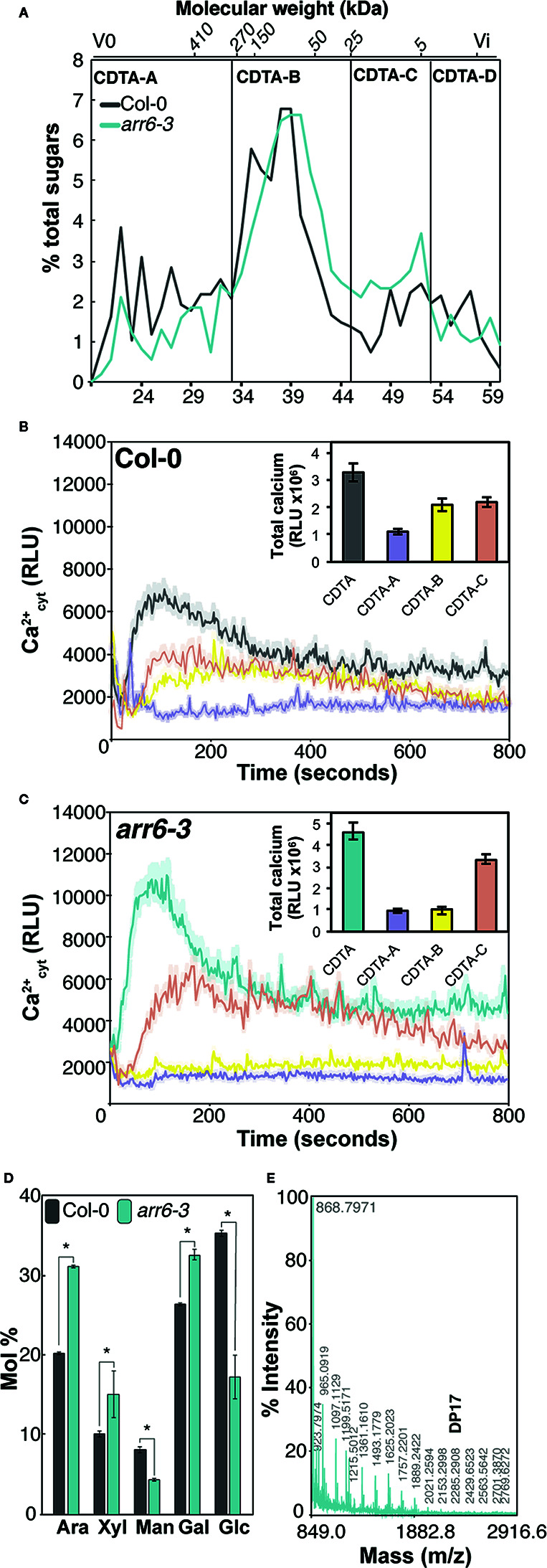
Pectin-CDTA sub-fractions between 25 and 5 kDa retain most of the activity triggering Ca^2+^ influxes and are enriched in pentose oligosaccharides. **(A)** Size exclusion chromatography (SEC) elution profile (Sepharose CL-6B) of pectin fractions (CDTA extract) from wild type (Col-0) and *arr6-3* plants. The Sepharose column was calibrated with commercial dextrans of known weight-average relative molecular mass and the elution fraction number (bottom) of some of them is indicated on the top of the chromatogram. The elution profiles were monitored by total sugar quantification (phenol-sulfuric method). Sub-fractions were defined as: [A] > 270 kDa, [B] 270–25 kDa, [C] 25–5 kDa, [D] <5 kDa. Profiles are representative of ten independent preparations. **(B, C)** Ca^2+^ influx kinetics triggered by CDTA sub-fractions A-C from Col-0 **(B)** and *arr6-3* **(C)** plants in Col-0^AEQ^ seedlings. Elevations of cytoplasmic calcium concentrations over 800 s were measured as relative luminescence units (RLU). Data are means (n=8) from one experiment representative of three independent ones with similar results. The total areas-under-the-curves were integrated and their average values ± SD (n=8) are represented at the right side of each panel. **(D)** Monosaccharide composition (Mol % ± SD, n=3) of Col-0, and *arr6-3* CDTA-C sub-fraction. Ara: arabinose; Xyl: xylose; Man: mannose; Gal: galactose; Glc: glucose. Statistically significant differences between genotypes according to Student’s t-test (*p < 0.05). **(E)** MALDI-TOF mass spectrum of CDTA-C sub-fraction. M/z shifts are coherent with the presence of pentose oligosaccharides of different degree of polymerization (DP). The spectrum shown (*arr6-3*) is representative of all analyzed pectin I-C sub-fractions (n≥10).

In a previous work, we hypothesized that the molecular basis of the differential disease resistance responses in the Arabidopsis *arr6-3* mutant allele could be associated with the enhanced and differential presence of carbohydrate-based DAMPs in the pectin-enriched fractions derived from their cell walls (Bacete et al., 2020). These DAMPs, when released, would activate immune responses, thus triggering disease resistance. Pectic fractions isolated from *arr6-3* cell walls triggered more intense Ca^2+^ influxes and MAPK phosphorylation than the fractions from wild-type plants (Bacete et al., 2020), thus they were selected for further analyses in order to characterize the putative DAMPs responsible for the observed differential immune responses in *arr6-3* plants. Pectin-enriched fractions from *arr6-3* and Col-0 plants, extracted with 1,2-cyclohexylenedinitrilotetraacetic acid (CDTA) from purified cell walls, were further fractionated by size exclusion chromatography to obtain samples containing carbohydrates with distinct molecular weights. Four sub-fractions (CDTA-A to CDTA-D) were obtained, containing molecules with different theoretical sizes: *i)* CDTA-A: >270 kDa; *ii)* CDTA-B: 270-25 kDa; *iii)* CDTA-C: 25-5 kDa; *iv)* CDTA-D: <5 kDa (**Figure 1A**). These masses are estimated as the Sepharose column was calibrated with commercial dextrans of known weight-average relative molecular mass, which may not display similar conformations as pectic polymers. Total sugar quantifications showed that even after long dialysis procedures, the CDTA-D fractions contained very low amounts of carbohydrates and were most likely composed of the solvent used to obtain this fraction (CDTA; Mort et al., 1991), and therefore, they were excluded for further analyses. CDTA-A, -B, and -C were tested for their capacity to trigger intracellular Ca^2+^ entry, an early immune response, in Col-0^AEQ^ sensor lines (Ranf et al., 2012). CDTA-C sub-fractions from both Col-0 and *arr6-3* retained most of the activity of the complete CDTA-pectin fractions (**Figures 1B, C**), whereas CDTA-A or -B from *arr6-3* did not present any activity and CDTA-B from Col-0 still presented some activity (**Figures 1B, C**). Thus, we concluded that potential active DAMPs were most abundant in the *arr6-3* CDTA-C sub-fractions.

Neutral sugar analyses by GC/MS revealed that CDTA-C sub-fractions were still very complex in terms of monosaccharide composition, challenging further predictions about the identity of novel DAMPs that they would contain (**Figure 1D**). However, the enrichment of *arr6-3* CDTA-C sub-fractions in arabinose and xylose (**Figure 1D**) was in line with MALDI-TOF/TOF mass spectrometry analyses, which showed only the presence of pentose oligosaccharides (m/z shifts of 132) with degree of polymerization (DP) up to 17 (**Figure 1E**). Other oligosaccharide signatures were not found in the MALDI-TOF/TOF, clearly indicating that pentose-containing carbohydrates could be novel DAMPs present in the PTI-active, CDTA-extractable pectin-enriched fractions of Arabidopsis. However, further fractionation of CDTA-subfractions resulted non-viable due to their complexity in terms of composition and polydispersity combined with the low yields obtained. Thus, we decided to investigate the capacity of different oligosaccharides containing arabinose and xylose which could be obtained from commercial AXs using specific glycosyl hydrolases (GH).

### Arabinoxylan Oligosaccharides With Different DP Trigger Calcium Influxes in Arabidopsis

**Figure 2:**
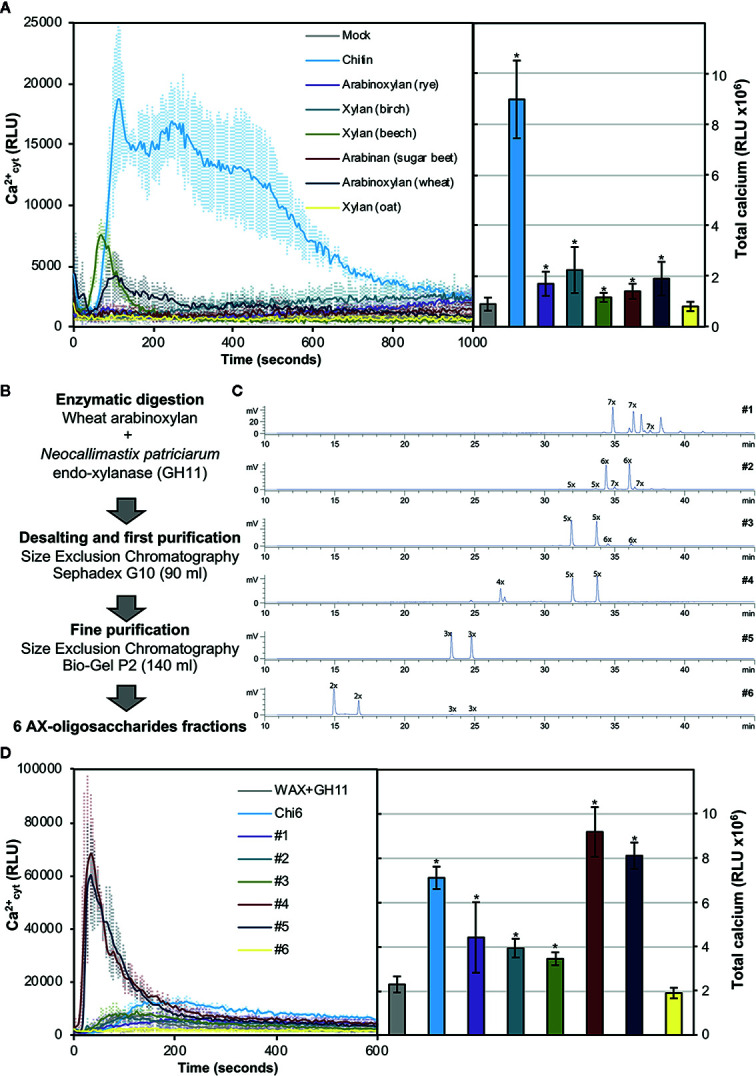
Pentose-based oligosaccharides trigger cytoplasmic calcium elevations. **(A)** Calcium influx measured as relative luminescence units (RLU) over the time in 8-d old Arabidopsis Col-0^AEQ^ seedlings after treatment with 0.5 mg/ml of chitin and arabino-xylan polysaccharide preparations. The total areas-under-the-curves were integrated and their values are represented at the right side of the panel. Data represent mean ± SD (n=8) from one experiment representative of three independent ones with similar results. Statistically significant differences according to Student’s t-test (*p<0.05) compared to negative control (mock) are shown. **(B)** Preparation and fractionation pipeline of pentose-based oligosaccharides from wheat arabinoxylan (AX). **(C)** HPLC-ELSD chromatograms of purified oligosaccharide preparations. Peaks are labeled as nX, where “n” correspond to the number of pentoses contained. Double peaks correspond to alpha-/beta-anomeric isomers at the reducing end of each detected oligosaccharide. **(D)** Calcium influxes after treatment with 0.5 mg/ml of the GH11-digested wheat AX before chromatographic purifications (WAX+GH11), chitohexaose (Chi6) and the purified pentose-based oligosaccharides (#1–6). The total areas-under-the-curves were integrated and their values are represented at the right side of the panel. Data represent mean ± SD (n=8) from one experiment representative of three independent ones with similar results. Statistically significant differences according to Student’s t-test (*p < 0.05) compared to negative control (WAX+GH11) are shown.

In order to investigate whether pentose-based structures could be sensed by Arabidopsis, we decided to analyze in a first instance the capacity to trigger Ca^2+^ influxes of different commercial polymeric structures (**Figure 2A**). As previously described, polysaccharides often need to be solubilized to smaller entities in order to trigger early immune responses in plants (Mélida et al., 2018). In this regard, partial solubilization by heating of water-dissolved polysaccharides can help to expose ligands which may not be accessible in their insoluble counterparts. Heat-solubilized xylan from beech and AX from wheat triggered subtle calcium influxes compared to chitin, but still represented good candidates as pentose-DAMP sources (**Figure 2A**). Based on these results, we selected wheat AX as the polymeric source to be hydrolyzed to oligosaccharides given that arabinose decorations could mean an advantage compared to non-decorated xylans when enzymatic hydrolysis is used to generate different oligosaccharides of desired DP (McCleary et al., 2015). Wheat AX was hydrolyzed with an endo-xylanase (GH11) from *Neocallimastix patriciarum* and 6 AX-oligosaccharide fractions (#1 to #6) were purified through two rounds of size exclusion chromatography (**Figure 2B**). Purified fractions contained pentose-oligosaccharides ranging from DP 2 to 9 as demonstrated by HPLC-ELSD and MS/MS (**Figure 2C** and **Supplementary Figure S1**). Interestingly, we found that Ca^2+^ burst in treated plants was activated by all fractions except #6, which contained mainly a disaccharide and minor amounts of a trisaccharide (**Figure 2D** and **Supplementary Figure S1**). Since fraction #5 also contained a trisaccharide but triggered intense Ca^2+^ influxes, it seems that a DP above 2 is required by Arabidopsis perception machinery in case of pentose-based oligosaccharides. Together with fraction #5, fraction #4 resulted the most active and according to HPLC-ELSD and MS/MS these contained pentose oligosaccharides ranging from DP 3 to 5 (**Figure 2D** and **Supplementary Figure S1**).

**Figure 3:**
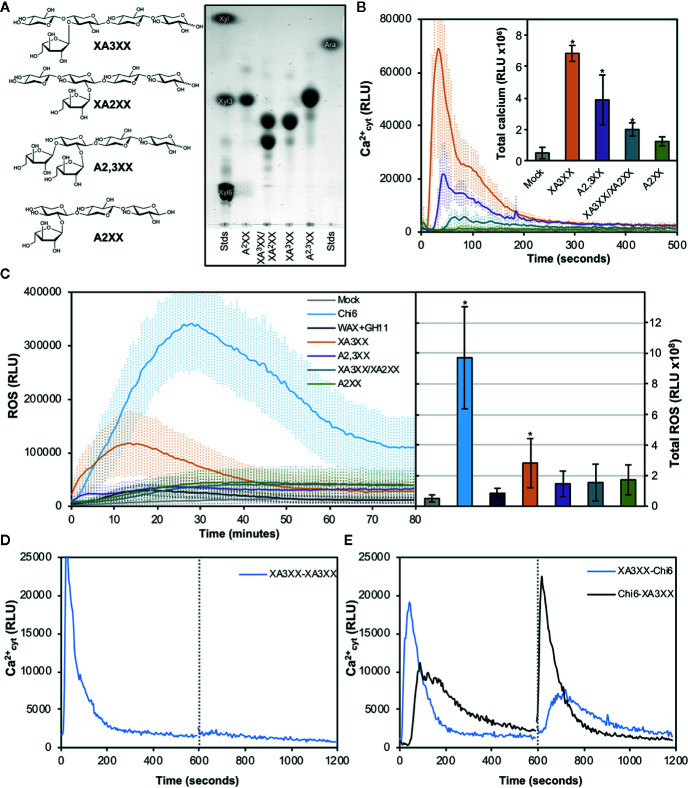
Pure arabinoxylan (AX) oligosaccharides trigger early immune responses in Arabidopsis. **(A)** Molecular structures of the different AX oligosaccharides used in the experiments. 3^3^-α-L-arabinofuranosyl-xylotetraose (XA3XX), 2^3^-α-L-arabinofuranosyl-xylotetraose (XA2XX), 2^3^,3^3^-di-α-L-arabinofuranosyl-xylotriose (A2,3XX), and 2^3^-α-L-arabinofuranosyl-xylotriose (A2XX). Thin layer chromatography profiles of the pure commercial AXs used in the experiments using 1-propanol/ethyl-acetate/water (9:7:4 by volume) as mobile phase. Left and right lanes show xylose (Xyl), xylotriose (Xyl3), xylohexaose (Xyl6) and arabinose (Ara) which were used as markers. TLC shown is from one run representative of three independent ones with similar results. **(B)** Calcium influx measured as relative luminescence units (RLU) over the time in 8-d-old Arabidopsis Col-0^AEQ^ seedlings after treatment with 500 μM of pure AX oligosaccharides. Water (mock) was used as negative control. The total areas-under-the-curves were integrated and their values are represented at the right side of the panel. Data represent mean ± SD (n=8) from one experiment representative of three independent ones with similar results. Statistically significant differences according to Student’s t-test (*p < 0.05) compared to negative control are shown. **(C)** Reactive oxygen species (ROS) production (by Luminol reaction) after treatment with 500 μM of pure AX oligosaccharides in Arabidopsis leaf-discs measured as RLU over the time. Water (mock), GH11-digested wheat AX before chromatographic purifications (WAX+GH11) and chitohexaose (Chi6; 100 μM) were used as negative (mock and GH11+WAX) and positive (Chi6) controls. The total areas-under-the-curves were integrated and their values are represented at the right side of the panel. Data represent mean ± SD (n=8) from one experiment representative of three independent ones with similar results. Statistically significant differences according to Student’s t-test (*p < 0.05) compared to water control are shown. **(D, E)** Cross elicitation during the refractory period of calcium signaling between XA3XX (250 μM) and chitohexaose (Chi6; 100 μM). Data show the elevation of cytoplasmic calcium concentration, measured as relative luminescence units (RLU), over the time in 8-d-old Arabidopsis Col-0^AEQ^ seedlings after treatments. Dashed line (600 s) indicates the application time of the second elicitor within the refractory period of the first elicitation. In **(E)**, blue line represents a first treatment of XA3XX followed by Chi6 after 600 s, while black line represents a first treatment with Chi6 followed by a second of XA3XX. Data represent mean (n=8) from one experiment representative of three independent ones with similar results.

**Figure 4:**
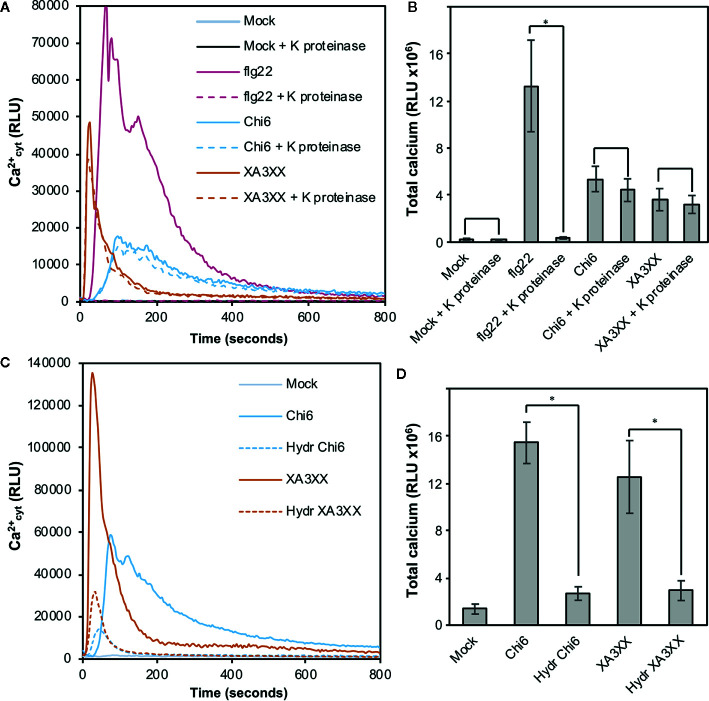
Proteolysis and acid hydrolysis effects on XA3XX calcium signaling. Variation of intracellular Ca^2+^ concentration after 800 s of treatment of Col-0^AEQ^ seedlings with untreated flg22 (1 μM), chitohexaose (Chi6; 100 μM), or XA3XX (250 μM) **(A, B)** with or without proteinase K previous digestion and **(C, D)** with or without acid hydrolysis previous digestion. Data represent mean ± SD (n=8) from one experiment representative of two independent ones with similar results. Statistically significant differences according to Student’s t-test (*p < 0.05).

In view of the results obtained with our purified oligosaccharides, we decided to investigate well-defined and highly-pure commercial structures which most likely resemble those from our purifications (McCleary et al., 2015). These included the pentasaccharides 3^3^-α-L-arabinofuranosyl-xylotetraose (XA3XX), 2^3^-α-L-arabinofuranosyl-xylotetraose (XA2XX), 2^3^,3^3^-di-α-L-arabinofuranosyl-xylotriose (A2,3XX), and the tetrasaccharide 2^3^-α-L-arabinofuranosyl-xylotriose (A2XX) (**Figure 3A**). Readouts from two early PTI events, such as Ca^2+^ influxes and production of reactive oxygen species (ROS), upon plant treatment with these oligosaccharides indicated that the different pentasaccharides tested were able to trigger immune responses on Arabidopsis seedlings and plants, the responses induced by XA3XX being the most intense ones (**Figures 3B, C**). Interestingly, cross-elicitation experiments, by sequential application of two compounds in 600 s interval, demonstrated that fractions #4 and #5 and commercial XA3XX had a refractory period of Ca^2+^ influx. Notably, this effect was not observed when the well characterized MAMP chitohexaose was used in the experiments (**Figures 3D, E** and **Supplementary Figure S2**). Although a refractory state does not necessarily indicate the same perception mechanism or receptor, these results indicated that these pentose oligosaccharides ranging from DP 3 to 5 have equivalent activities and, at least in Arabidopsis, differ from chitin-based signaling. According to manufacturer XA3XX is a pure carbohydrate, but given the low required doses of peptide MAMP/DAMPs to be perceived by plants (Stegmann et al., 2017), we performed proteinase K proteolytic digestions of XA3XX solutions, and of two MAMPs solutions (chitohexaose and flg22), used as controls (**Figures 4A, B**). Of note, both carbohydrate-based elicitors remained fully active in the Ca^2+^ system after proteolytic treatment, which contrasted with the immune activity abolishment observed in case of flg22 after proteinase K treatment (**Figures 4A, B**). On the other hand, in order to confirm that XA3XX elicitor activity was linked to its oligomer structure, acid hydrolysis of the oligosaccharide was performed. Our results indicated that the hydrolyzed XA3XX lost its capacity to trigger Ca^2+^ influxes as it was the case for chitohexaose (**Figures 4C, D**). All these data, together with the observation that XA3XX was also able to trigger a ROS burst in soybean plants (**Supplementary Figure S3**), clearly indicated that this pentasaccharide deserved a more detailed investigation.

### Arabinofuranosyl-Xylotetraose (XA3XX) Activates Several PTI Hallmarks Through a Novel Plant Sensing Mechanism

**Figure 5:**
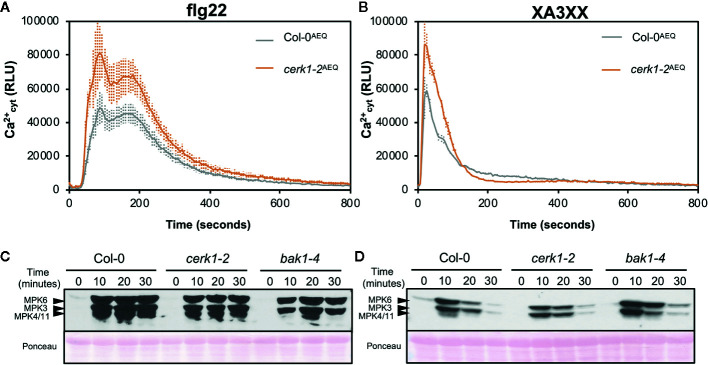
Pattern-triggered immunity hallmarks comparison between flg22 and arabinofuranosyl-xylotetraose (XA3XX). Flg22 **(A, B)** and XA3XX **(C, D)** final concentrations were 1 and 500 μM respectively. **(A, B)** Elevations of cytoplasmic calcium concentrations over time in 8-d-old Arabidopsis Col-0^AEQ^ and *cerk1-2* ^AEQ^ seedlings upon treatments. Data represent mean ± SD (n=8) from one experiment representative of three independent ones with similar results. **(C, D)** MAPK activation in 12-d-old Arabidopsis seedlings of the indicated genotypes. The phosphorylation of MPK6, MPK3, and MPK4/MPK11 was determined by Western blot, using the anti-pTEpY antibody, at the indicated time points (minutes) after treatment. Ponceau red-stained membranes show equal loading. Western-blot shown is from one experiment representative of three independent ones with similar results.

In order to further characterize the early immune responses triggered by XA3XX, we performed a more detailed analysis of the Ca^2+^ kinetics following the application of the pentasaccharide and the peptide MAMP flg22 (**Figures 5A, B**). Flg22 induced a double Ca^2+^ burst peak at about 90 and 180 s followed by a maintained decrease in luminescence that lasted about 600 s (**Figure 5A**). However, XA3XX kinetics was very different, with a very fast single peak at 20 s post-application and a rapid signal lost at about 200 s (**Figure 5B**). In Arabidopsis, lysin motif-(LysM)-PRR CERK1 (Chitin Elicitor Receptor Kinase 1) plays a central role as a co-receptor for several glycan MAMPs such as chitin, peptidoglycan a β-1,3-glucans (Willmann et al., 2011; Liu et al., 2012; Mélida et al., 2018). The use of *cerk1-2* mutant aequorin lines demonstrated that the perception of XA3XX, like that of flg22, was CERK1-independent, which was in line with the refractory experiments with XA3XX/chitin (**Figures 5A, B**).

Next, we monitored phosphorylation of downstream protein kinases (MPK3/MPK6/MPK4/MPK11), a PTI hallmark, in Col-0 wild-type plants and *cerk1-2* and *bak1-4* mutants (in Col-0 background) impaired in PRR co-receptors required for the perception of chitin and flg22, respectively (**Figures 5C, D**). These analyses confirmed that XA3XX recognition by Arabidopsis plants was CERK1-independent and demonstrated that it was also BAK1-independent, while a partial BAK1-dependence for flg22 was observed, as described (Chinchilla et al., 2007). Western-blot assays confirmed MPK3- and MPK6-phosphorylation after application of a XA3XX solution to Arabidopsis seedlings, reaching the highest level of phosphorylation at 10 min post-treatment (**Figure 5D**). MPK4/11-phosphorylation was almost not-detectable in XA3XX-treated plants. MPKs phosphorylation levels of plants treated with XA3XX was weaker than phosphorylation of MPK3/MPK6/MPK4/MPK11 at all time points tested after elicitation with flg22 (**Figure 5C**).

**Figure 6:**
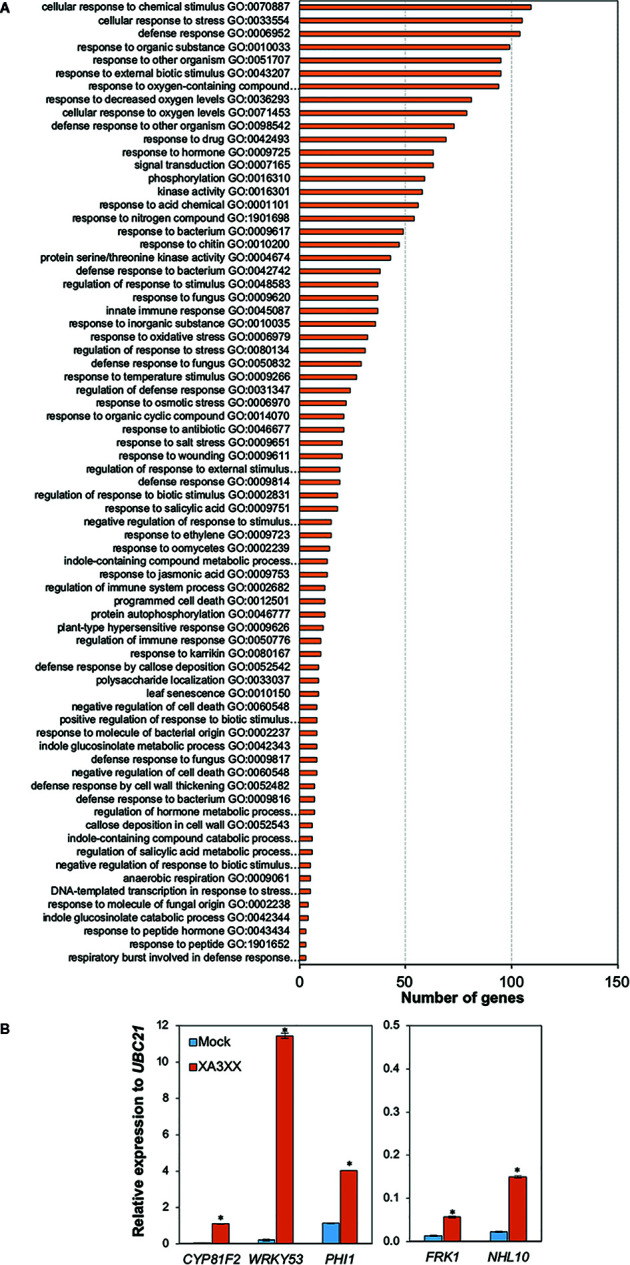
Functional classification of arabinofuranosyl-xylotetraose (XA3XX)-differentially expressed genes in Arabidopsis. **(A)** Biological process Gene Ontology (GO) term enrichment map of the 460 overexpressed genes in 12-d-old Arabidopsis Col-0 plants at 30 min after 250 μM XA3XX treatment. GO term enrichment is expressed as number of mapped genes. Data used to build the histogram can be retrieved from **Supplementary Table S2**. **(B)** RNA-seq data validation by quantitative RT-PCR analysis in 12-d-old Arabidopsis seedlings. Relative expression levels to *UBC21* (*At5g25769*) gene at 30 min are shown. Data represent mean ± SD (n=3) from one experiment representative of two independent ones with similar results. Statistically significant differences between XA3XX and mock according to Student’s t-test (*p < 0.005).

Global gene reprogramming is the expected output of earlier PTI events such as Ca^2+^ influxes, ROS production and MAPK phosphorylation. Such alteration in the expression patterns of specific genes would determine the adaptation ability of a given plant to respond to a potential infection by pathogens. To further characterize the basis of XA3XX-mediated immunity, we performed RNA-seq analyses of Col-0 seedlings treated for 30 min with XA3XX (**Figure 6** and **Supplementary Tables S1** and **S2**). Elicitation with XA3XX changed the expression of 511 genes, most of which (460) were up-regulated (**Supplementary Table S1**). XA3XX up-regulated genes mainly grouped into gene ontology (GO) terms related to innate immune and defense response to different stimuli, kinase and signal transduction activities, and indole-containing compound metabolic processes (**Figure 6A**), further corroborating the function of XA3XX in modulating PTI. We validated RNA-seq data of five PTI-marker genes (*CYP81F2*, *WRKY53, PHI1, FRK1*, and *NHL10*) by qRT-PCR in seedlings 30 min after treatment, and we found that all these genes were up-regulated after XA3XX elicitation compared to mock-treated seedlings (**Figure 6B**) confirming a full PTI response of Arabidopsis plants treated with XA3XX. Together, these analyses suggest that XA3XX-induced responses are addressed to a global immune response.

### XA3XX Crop Pre-Treatment Diminishes Pathogen Disease Symptoms Through a Non-Yet Characterized PRR Complex

**Figure 7:**
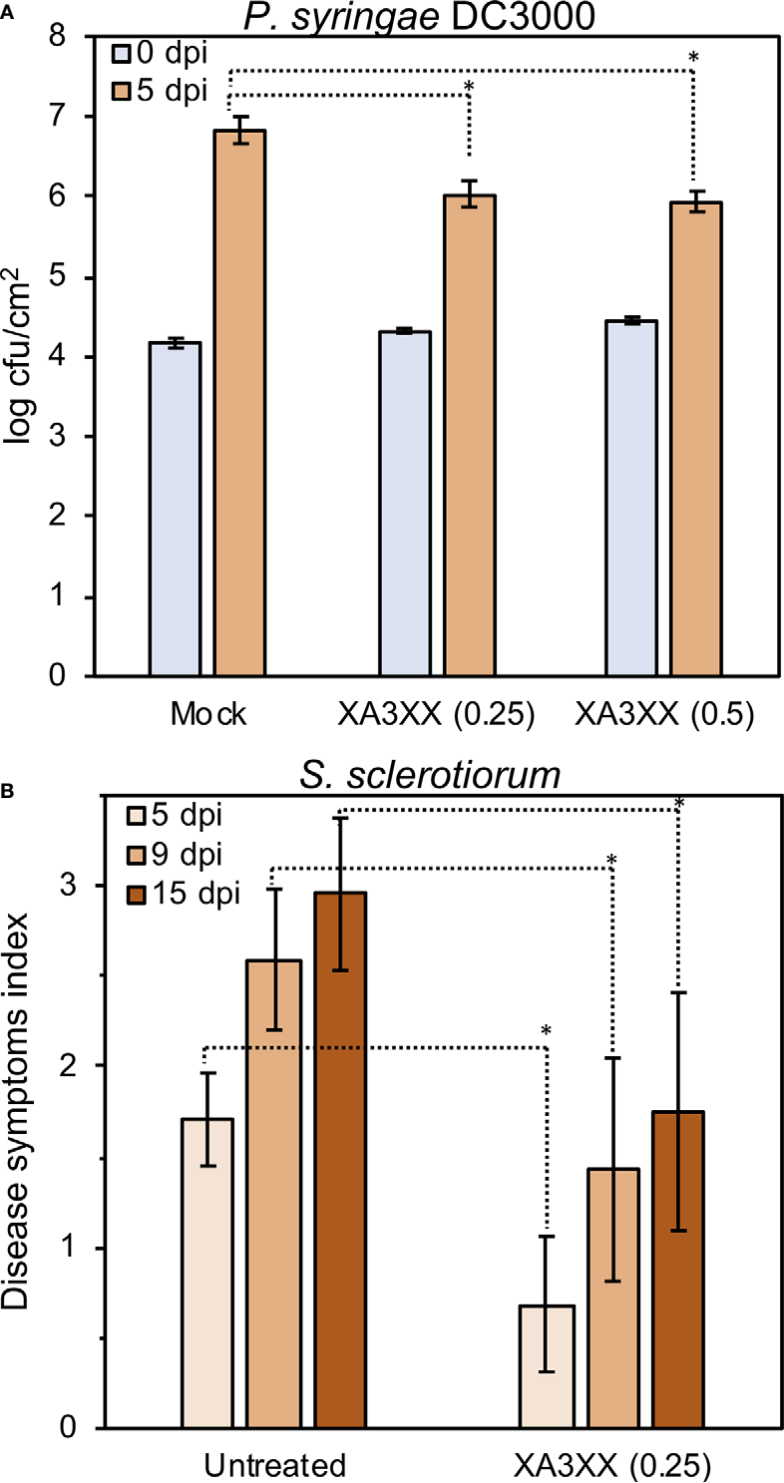
Treatment of tomato and pepper plants with arabinofuranosyl-xylotetraose (XA3XX) confers enhanced disease resistance to pathogens. Plants were foliar-treated with XA3XX (0.25–0.5 mg/plant) 2 d prior bacterial or fungal inoculation. **(A)** Colony forming units (cfu) of *Pseudomonas syringae* pv*. tomato* DC3000 per leaf area (cm^2^) at 0- and 5-d post-inoculation (dpi) in three-week-old tomato plants. Cfu/cm^2^ were determined after plating serial bacterial dilutions obtained from tomato leaf discs of known area onto KB plates. Data represent mean ± SD (n=8). **(B)** Disease symptoms index produced by *Sclerotinia sclerotiorum* at 5-, 9-, and 15-dpi in leaves of pepper plants. Data represent mean ± SD (n=24). Statistically significant differences according to Student’s t-test (*p < 0.05).

Exposure of plants to active MAMP/DAMPs prior to subsequent pathogen attack may allow a more efficient plant defense activation through PTI activation (Héloir et al., 2019; Schellenberger et al., 2019). We showed that XA3XX was perceived by Arabidopsis and soybean (**Figure 3** and **Supplementary Figure S3**). Therefore, we tested the elicitor7nbsp;activity of XA3XX in three-week-old tomato plants (Moneymaker) treated by foliar spray with XA3XX 2 d before inoculation with the biotroph *Pseudomonas syringae* pv *tomato* DC3000 (10^8^ cfu/ml). Notably, bacterial population, determined as colony forming units (cfu) per leaf area, was significantly reduced in tomato XA3XX-pretreated plants compared to mock-treated plants (**Figure 7A**). Bacterial growth reduction found at 5 d post-inoculation (dpi) were in the order of 0.8–0.9 log of cfu/cm^2^ when 0.25 and 0.5 mg of XA3XX per tomato plant were applied as pre-treatment, respectively (**Figure 7A**). Previous studies using similar approaches have also shown protection results of carbohydrate-based DAMPs against fungal necrotrophs (Claverie et al., 2018). We next tested XA3XX-pre-treated pepper plants against the necrotroph fungi *Sclerotinia sclerotiorum*. Treated pepper plants showed a reduction in the disease symptoms index at 5, 9, and 15 dpi in comparison to control plants (**Figure 7B**). These data indicate that XA3XX is able to trigger immune responses in some dicot crops conferring disease resistance.

## Discussion

Plant pathogens and their hosts have co-evolved an arsenal of CWDE to break-down the opponent’s cell wall during the interactions (Rovenich et al., 2016). Thus, plant and microbial cell walls are rich sources of carbohydrate-based defense signaling molecules (DAMPs/MAMPs), that are under-characterized. Recently, we have showed that impairment of *ARR6* gene in Arabidopsis affects cell wall composition, which may lead to the accumulation of DAMPs that would favor a “defense-ready” state, thus affecting plant-pathogen interactions (Bacete et al., 2020). Remarkably, pectin-enriched cell wall fractions extracted from *arr6* cell walls resulted to be enriched in carbohydrate-based DAMPs compared to wild-type fractions. However, the composition of such DAMPs could not be deciphered. In this work we have attempted to unveil the nature of these DAMPs. Unexpectedly, analytical data obtained from size-exclusion chromatography purifications suggested, for the first time, that pentose-based oligosaccharides co-extracted with pectins (using calcium chelators) could play a role as plant DAMPs. Next, we asked whether pentose-based oligosaccharides could be a novel group of plant DAMPs, and indeed we have demonstrated here that AXs can be perceived as molecular patterns by plants. In particular, we identified several active oligosaccharides structures, with pentasaccharide XA3XX being the most active one (**Figure 3**). In particular, XA3XX triggers Ca^2+^ influxes, ROS production, MAPK phosphorylation, and a global gene reprogramming in Arabidopsis at micromolar concentrations. Bearing in mind that the presence of glucuronoAXs has been suggested to be a component of Arabidopsis cell walls (Zablackis et al., 1995), XA3XX and related structures characterized in this work could be considered as plant DAMPs even in Arabidopsis model species.

Structurally, the most similar plant DAMPs characterized so far would be other β-1,4-linked hemicelluloses such as xyloglucan and mannan (Claverie et al., 2018; Zang et al., 2019). Xyloglucan recently proposed as DAMP consists of a β-1,4-glucan backbone associated with xylosyl, galactosyl, and fucosyl-type branching, mainly of DP 7 (Claverie et al., 2018). The purified xyloglucan triggered MAPK phosphorylation and immune-associated gene expression in Arabidopsis and *Vitis vinifera*, but no ROS production was found (Claverie et al., 2018). In another recent work, Zang et al., produced mannan oligosaccharides (DP 2–6) by enzymatic hydrolysis of locust bean gum and demonstrated their DAMP potential on *Nicotiana benthamiana* and *Oryza sativa* (Zang et al., 2019). Mannan oligosaccharides triggered Ca^2+^ influxes, ROS production, stomata closure, and over-expression of defense-related genes such as *PR-1a* and *LOX*. Interestingly, these novel groups of hemicellulosic DAMPs, including xyloglucans, mannans, and AXs display the same type of glycosidic linkage in their backbone (β-1,4-linked) as the previously characterized plant DAMPs pectic OGs and cello-oligosaccharides (Aziz et al., 2007; Ferrari et al., 2013; Benedetti et al., 2015; Souza et al., 2017; Johnson et al., 2018). In contrast to cello-oligomers (DAMP) and chitin oligosaccharides (MAMP), which are actively triggering broad immune responses in the low micromolar range, the rest of glycan cell wall derived DAMPs characterized (including XA3XX) are only active at high micromolar concentrations, although the activities of the DAMPs/MAMPs are difficult to compare since experiments were performed in different labs, using different experimental setups and species (Aziz et al., 2007; Claverie et al., 2018; Johnson et al., 2018; Mélida et al., 2018; Zang et al., 2019; and this work). It should also be noted that cell wall polysaccharides can be very abundant in plant cells and they could yield very high concentrations of their main components, such as cellulose and xylans (over 5% of Arabidopsis fresh weight; Sakamoto and Mitsuda, 2015), and of the DAMPs derived from these polymers. Anyway, in spite of the high doses required of these novel groups of hemicellulosic DAMPs to be perceived by plants as such, they were able to enhance plant protection against different plant pathogens. Xyloglucan effectively protected grapevine and Arabidopsis against the necrotrophic fungus *Botrytis cinerea* or the oomycete *Hyaloperonospora arabidopsidis* pathogens while mannans improved rice protection against the bacteria *Xanthomonas oryzae* and the oomycete *Phytophthora nicotianae*, respectively (Claverie et al., 2018; Zang et al., 2019). In this work we have shown the protection capacity of XA3XX on tomato and pepper plants against bacterial plant pathogen *P. syringae* and the fungus *S. sclerotiorum* (**Figure 7**).

Xylans are main hemicelluloses of dicot secondary cell walls whose presence is essential for plant development, as exemplified in Arabidopsis plants with reduced xylan quantity, which show weakened cell walls and are unable to develop a vascular system (Brown et al., 2007; Wu et al., 2009). The importance of xylans in plant resistance to pathogens has been suggested previously, though the molecular bases of xylan-associated resistance phenotypes were largely unknown. For example, Arabidopsis plants with enhanced levels of xylose in their cell walls, as it occurs in Arabidopsis *de-etiolated3* (*det3*) and *irregular xylem6* (*irx6*) mutants (Brown et al., 2005; Rogers et al., 2005) or with modifications in their xyloglucan structure, as it is the case of the Arabidopsis *xyl1-2* mutant (Sampedro et al., 2010), show an enhanced resistance to the necrotrophic fungus *Plectosphaerella cucumerina* (Delgado-Cerezo et al., 2012). In contrast, Arabidopsis *er* plants, impaired in ERECTA Receptor-Like Kinase, and *agb1* and *agg1 agg2* mutants, impaired in the Gβ and Gγ subunits of heterotrimeric G proteins, that are hypersusceptible to the same necrotrophic fungus, show a reduced xylose content (Llorente et al., 2005; Sánchez-Rodríguez et al., 2009; Delgado-Cerezo et al., 2012). Also, alteration of cell wall xylan acetylation caused by Arabidopsis ESKIMO1 impairment was shown to enhance plant disease resistance to several pathogens, including *P. cucumerina* (Escudero et al., 2017). Whether these modifications in cell wall xylans are linked to an enhanced pentose-based DAMPs release from weakened walls (increased resistance) or to the alteration of pathogen capability to penetrate host tissues upon secretion of their CWDE repertories are two interesting questions to address in future works. CWDE able to hydrolyze xylan polysaccharides to DAMPs such as those described in this work, are endo-1,4-β-xylanases belonging to GH families 10 and 11 (McCleary et al., 2015). In particular, studies on GH11 β-xylanases crystal structures, such as that from *N. patriciarum* used in this work, showed that the α-L-Ara*f* can be accommodated on O-2 and O-3, thus being able to release structures such as XA3XX and XA2XX (Vardakou et al., 2008). However, Arabidopsis only displays a handful of GH10 endo-xylanases in its genome, but any GH11 (see CAZy database at www.cazy.org/e1.html). GH10 endo-xylanases would cleave arabinose-decorated non-reducing ends instead of xylose-free ones as is the case of XA3XX (Suzuki et al., 2002; McCleary et al., 2015). Therefore, our hypothesis is that the activity of GH10 and GH11 xylanases from pathogens might be associated to the release of xylan-derived DAMPs such as XA3XX and that such release might be differential in cell wall mutants displaying a modified architecture which could make some structures more or less accessible to GHs from pathogens, that could be the case of *arr6* mutants (Bacete et al., 2020). Indeed, bacterial and fungal endo-1,4-β-xylanases have been shown to be required for full virulence of plant pathogens such as *B. cinerea* and *Xanthomonas* (Brito et al., 2006; Santos et al., 2014).

Notably, we show here that XA3XX is perceived by dicot crops, like soybean, tomato, and pepper, supporting that other plant species than Arabidopsis harbor the mechanisms required to perceive xylan-derived DAMPs. This perception, at least in the case of Arabidopsis, is independent of the co-receptors CERK1 and BAK1, further indicating that the mechanism of AX perception differs from that of chitin and β-1,3-glucans (Liu et al., 2012; Cao et al., 2014; Mélida et al., 2018). Taken together that arabinofuranose substitutions are less frequent in dicot than in monocot xylans and that these hemicelluloses are quite more abundant in the monocot branch, it will be interesting to test whether these and related pentose-based DAMPs trigger stronger or lower (if any) responses in plant species at different phylogenetic positions than those included in this study (all dicots). Regarding MAMPs, only a minor fraction of them (flg22, peptidoglycan, and chitin) are recognized by PRRs that are widespread among plants and can be found in both monocot and dicot plant species (Albert et al., 2020). Sensor systems for such patterns are considered an ancient set of PRRs, however the majority of PRRs known to date exhibit genus-specific distribution patterns. On the other hand, PRR-independent perception mechanisms for carbohydrate-based DAMPs could have been evolutionary selected. Therefore, future work in the characterization of the perception mechanisms and the specific immune pathways triggered by AXs in different species will be necessary to unveil their functions and to determine if this is part of an additional mechanism of inter plant species recognition. Our findings support the use of carbohydrate-based DAMPs/MAMPs as biological products for the regulation of crops immunity and disease resistance responses. The use of these biological products in agriculture production can contribute to reach the social demand of a more sustainable agriculture.

## Materials and Methods

### Biologic Material and Growth Conditions

All Arabidopsis lines used in this study were in the Columbia-0 (Col-0) background. Arabidopsis plants used for [Ca^2+^]_cyt_, MAPKs and gene expression analyses were grown in 24-well plates (10 seedlings per well) under long day conditions (16 h of light) at 20–22°C in liquid MS medium [0.5x Murashige & Skoog basal salt medium (Duchefa), 0.25% sucrose, 1 mM MES, pH 5.7]. Soil-grown Arabidopsis plants used for cell wall isolation and ROS assays were maintained under short day conditions (10 h of light). Tomato (*Solanum lycopersicum*, Moneymaker), pepper (*Capsicum annuum*, Murano), and soybean (*Glycine max*, Annushka) plants were grown in soil under greenhouse conditions.

### Statistical Methods

As a general rule, data shown are means ± standard deviation (SD) from a given number of replicates (n≥3). Data was normally retrieved from one representative independent out of three, however, given the particularity of each assay specific details are indicated in figure footnotes and in specific method subsections below. Asterisks indicate significant differences according to Student’s t-test analysis, * *p* ≤ 0.05 (R software).

### Carbohydrates

Details about carbohydrates used in this work can be found in **Supplementary Table S3**. AX polysaccharides (from wheat and rye), oligosaccharides (XA3XX, XA2XX, XUXX, A23XX, A2XX), and chitohexaose (β-1,4-D-(GlcNAc)_6_; Chi6) were purchased from Megazyme. Xylan (from birch, beet, and oat) and arabinan (from sugar beet) were purchased from Sigma-Aldrich.

### Arabidopsis Cell Wall Fractionation

Three-week-old Arabidopsis plants (n>50) were collected and immediately frozen in liquid nitrogen. Cell walls and their fractions were prepared as previously described (Bacete et al., 2017). The pectin-I fractions were size-fractionated by size-exclusion chromatography on Sepharose CL-6B (GE Healthcare, 140 ml bed-volume in a 1.6 cm diameter column) in 0.33 M sodium acetate buffer (pH 5.0). The column was connected to a Biologic-LP instrument (Bio-Rad) and the flow rate was 1.8 ml/min. The Sepharose column was calibrated with commercial dextrans (Sigma) of known weight-average relative molecular mass. Resulting sub-fractions were dialysed (Spectra/Por MWCO 1000 Daltons, Repligen) against deionized water to remove solutes of a small molecular mass (dialysis tubings were thoroughly washed before use to eliminate any contaminants potentially associated to the membranes). The entire process was repeated three times.

### Xylan Enzymatic Digestion and Oligosaccharides Purification

Five hundred mg of low viscosity wheat flour AX (P-WAXYL, Ara : Xyl 38:62) were added to 24.5 ml of deionized water at 60°C and dissolved by stirring on a magnetic stirrer until complete dissolution. Then, the solution was equilibrated to 40°C and 0.5 ml of 0.5 M sodium phosphate buffer, pH 6, were added. This solution was placed in a water bath at 40°C and 97.5 U of endo-1,4-β-D-xylanase from *Neocallimastix patriciarum* (Megazyme #E-XYLNP) were added and incubated at 40°C for 16 h. Reactions were terminated by incubating the solutions at 95°C for 5 min. Solutions were centrifuged at 9,400 *g* for 10 min to remove insoluble materials. Digestion products were freeze-dried, desalted and pre-purified using a Sephadex G-10 column (90 cm^3^ bed-volume in a 1.5 cm diameter column; Merck) and size-fractionated using a Biogel P2 Extrafine column (140 cm^3^ bed-volume in a 1.6 cm diameter column; BioRad). Columns were connected to a Biologic-LP instrument, distilled water was used as mobile phase and the flow rates were 0.24 ml/min. The entire process was repeated three times.

### Carbohydrate Analysis

The dried purified cell wall fractions (0.5 mg) were hydrolyzed in the presence of 2 M trifluoroacetic acid (TFA) at 121°C for 3 h. *Myo*-inositol was used as an internal standard. The resulting monosaccharides were converted to alditol acetates (Albersheim et al., 1967). Derivatized monosaccharides were separated and analyzed by gas chromatography (GC) on a SP-2380 capillary column (30 m x 0.25 mm i.d.; Supelco) using a Scion 450-GC system equipped with EVOQ triple quadrupole (Bruker). The temperature programme increased from 165°C to 270°C at a rate of 2°C min^-1^. MALDI-TOF MS analyses were performed using a 4800 Plus Proteomics Analyzer MALDI-TOF/TOF mass spectrometer (Applied Biosystems, MDS Sciex) as described (Mélida et al., 2018). Technical replicates were considered from the TFA hydrolysis step of a given cell wall fraction from the same extraction procedure.

Purified oligosaccharides were monitored by thin layer chromatography (TLC) and high-performance liquid chromatography (HPLC). Spotted-AXs were run twice on TLC Silicagel 60 plates (Merck) using 1-propanol/ethyl-acetate/water (9:7:4 by volume) as mobile phase. TLC plates were developed by dipping in a solution of 0.5% (w/v) thymol and 5% (v/v) H_2_SO_4_ in 96% (v/v) ethanol and heated at 80 °C for 5–8 min. The HPLC-ELSD analysis was performed as previously described (Senf et al., 2017). The oligosaccharides were injected into an Agilent 1200 Series HPLC equipped with an Agilent 6130 quadrupole mass spectrometer (MS) and an Agilent 1200 Evaporative Light Scattering Detector (ELSD). The purified oligosaccharides were separated on a graphitized carbon Hypercarb column (150 x 4.6 mm, Thermo Scientific) using a water (including 0.1% formic acid)-acetonitrile (ACN) gradient. The peaks in the ELSD traces were assigned based on their retention time and the corresponding masses in the MS. For additional MS analyses, a fraction of each oligosaccharide sample was injected directly into an Agilent 1260 Infinity II Series, LC/MSD XT (Single Quadrupol mit ESI-Jetstream-source).

### Aequorin Luminescence Measurements

Arabidopsis 8-d-old liquid-grown transgenic seedlings of ecotype Col-0 carrying the calcium reporter aequorin (Col-0^AEQ^; Ranf et al., 2012) were used for cytoplasmic calcium ([Ca^2+^]_cyt_) measurements using the method previously described (Bacete et al., 2017). Negative controls (water) were included in all the experiments. Aequorin luminescence was recorded with a Varioskan Lux Reader (Thermo Scientific). Data shown represent mean ± SD (n=8 seedlings) from one experiment representative of at least three independent ones with similar results.

### Reactive Oxygen Species

Five-week-old Arabidopsis or 6-week-old soybean plants were used to determine ROS production after treatments using the luminol assay (Escudero et al., 2017) and a Varioskan Lux luminescence reader (Thermo Scientific). Data shown represent mean ± SD (n=8 leaf discs from at least 4 different plants) from one experiment representative of three independent ones with similar results.

### Immunoblot Analysis of MAPK Activation

Twelve-day-old seedlings (n=10) grown on liquid MS medium in 24-well plates were treated with water (mock) and oligosaccharides for 0, 10, 20, and 30 min, and then harvested in liquid nitrogen. Protein extraction and detection of activated MAPKs using the Phospho-p44/42 MAPK (Erk1/2) (Thr202/Tyr204) antibody (Cell Signaling Technology) were performed as described (Ranf et al., 2011). Western-blot shown is from one experiment representative of three independent ones with similar results.

### Gene Expression Analyses

For gene expression analysis (qRT-PCR and RNA sequencing), 12-d-old seedlings grown on liquid MS medium were treated with the oligosaccharide or water (mock) solutions for 0 and 30 min. Total RNA was purified with the RNeasy Plant Mini Kit (Qiagen) according to the manufacturer’s protocol. qRT-PCR analyses were performed as previously reported (Delgado-Cerezo et al., 2012). *UBC21* (*At5g25760*) expression was used to normalize the transcript level in each reaction. Oligonucleotides used for detection of gene expression are detailed on **Supplementary Table S4**. Analysis of mock-treated seedlings showed no alterations in the expression levels of the marker genes used in this study. Data shown represent mean ± SD (n=3) from one experiment representative of two independent ones with similar results.

For RNA-seq analyses, samples from three biological replicates for each treatment were sequenced using 50bp Illumina Hiseq 2500. RNA-seq read raw data can be retrieved from the NCBI Sequence Read Archive (SRA) under BioProject accession ID PRJNA639010 (http://www.ncbi.nlm.nih.gov/bioproject/639010). Transcripts obtained were aligned against Arabidopsis annotation Araport11 (Cheng et al., 2017) using Hisat2 2.10.0 release (Kim et al., 2019). Afterwards, they were processed using Stringtie v1.3.6 (Pertea et al., 2015) and Ballgown R packages (Frazee et al., 2015) as previously described (Pertea et al., 2016). Differential expression analysis was performed with FPKM (Fragments Per Kilobase of transcript per Million mapped reads) values from the treatment against FPKM mock values in order to obtain the n-fold. For the up-regulated genes, a coverage cutoff of 50% of the dataset was applied to the treatment genes while for the down-regulated genes it was applied to the mock genes. N-fold of above or equal than 2 was used to prove up-regulation and an n-fold below or equal than 0.5 was applied to look for down-regulated genes. ClueGO 2.5.6 app for Cytoscape (Bindea et al., 2009) was used to determine which Gene Ontology (GO) categories were statistically overrepresented in the differentially expressed set of genes. Significant enrichments were determined using the Enrichment/Depletion (Two-sided hypergeometric) test and Bonferroni step down corrected *p* values are represented. Additional parameters are detailed in **Supplementary Table S2**.

### Crop Protection Assays

Three-week-old tomato plants (*Solanum lycopersicum*, Moneymaker) were sprayed with 2 ml of a XA3XX solution (0.125 or 0.25 mg/ml) containing 2.5% UEP-100 (Croda) and 2.5% Tween 24 MBAL (Croda) as adjuvants. Adjuvant solution was used as mock. *Pseudomonas syringae* pv. *tomato* DC3000 infections were performed 48 h after pre-treatments according mainly to Santamaría-Hernando et al., 2019. Briefly, plants were sprayed with a suspension of the bacterium (10^8^ cfu/ml) and two tomato leaf discs were collected from four different plants at 0- and 5-d post-infection (dpi). Colony forming units (cfu) per foliar area (cm^2^) were determined after plating serial bacterial dilutions obtained from tomato leaf discs of known area onto KB plates with rifampicin (25 µg/ml). Data shown represent mean ± SD (n=8) from one experiment representative of three independent ones with similar results. For *Sclerotinia sclerotiorum* experiments, 5-weeks-old pepper plants (*Capsicum annuum*, Murano) were treated using 5 ml of a XA3XX solution (0.05 mg/ml) containing 0.5% UEP-100 and 0.05% Tween 24 MBAL as adjuvants. Two-days after treatment, plants were moved to a 75% humidity greenhouse chamber and spray-inoculated with 5 ml of a 250 cfu/ml suspension of *S. sclerotiorum* homogenized mycelia according to Chen and Wang (2005). Disease symptoms were determined at 5 and 9 dpi in all the leaves of each plant (n=24) using a scale from 0 to 4 where 0 = no symptoms; 1 = little necrotic spots (< 20% of leaf area); 2 = two or more notable necrotic spots (20–50% of leaf area); 3 = more than 50% of leaf area affected, 4 = leaf senescence. Data shown represent mean ± SD (n=24) from three experiments.

## Data Availability Statement

RNA-seq read raw data can be retrieved from the NCBI Sequence Read Archive (SRA) under BioProject accession ID PRJNA639010.

## Author Contributions

HM and AM initiated, conceived and coordinated all the experiments. HM performed most of the experiments with support of GL, LB, and DR. IH performed the RNA-seq data analysis. CR and FP performed the HPLC-ELSD and MS experiments. HM prepared figures and tables. HM and AM wrote the paper with contributions of all the authors. All authors contributed to the article and approved the submitted version.

## Funding

This work was supported by grants IND2017/BIO-7800 of the Comunidad de Madrid Regional Government, BIO2015-64077-R of the Spanish Ministry of Economy and Competitiveness (MINECO), RTI2018-096975-B-I00 of Spanish Ministry of Science, Innovation and Universities, to AM. This work has been also financially supported by the “Severo Ochoa Programme for Centres of Excellence in R&D” from the Agencia Estatal de Investigación of Spain (grant SEV-2016-0672 (2017-2021) to the CBGP). In the frame of this program HM was supported with a postdoctoral fellow. DR was the recipient of an Industrial PhD Fellow (IND2017/BIO-7800) and IH was the recipient of an PhD FPU fellow from the Spanish Ministry of Education (FPU16/07118). FP thanks the Max Planck Society and the German Research Foundation (DFG, Emmy Noether program PF850/1-1 to FP) for financial support.

## Conflict of Interest

Authors DR and FB were employed by the company PlantResponse Biotech S.L.

The remaining authors declare that the research was conducted in the absence of any commercial or financial relationships that could be construed as a potential conflict of interest.
