# Supplementary material for: Arabinoxylan-Oligosaccharides Act as Damage Associated Molecular Patterns in Plants Regulating Disease Resistance
Source: Front Plant Sci. 2020 Aug 7;11:1210. doi: 10.3389/fpls.2020.01210 (PMC7427311; doi:10.3389/fpls.2020.01210)
Supplement: Supplementary file 1 [file DataSheet_1.pdf]

Supplementary Table S1: Differentially expressed genes under treatment with XA3XX in Arabidopsis

Expression level of the 511 differentially expressed genes when treating Col-0 plants with MLG43 and comparing with genes in Col-o mock treated plants

	Up-regulated genes in the treatment. Consideration of n-fold above 2 or equal to is taken
	Down-regulated genes in the treatment. Consideration of n-fold below or equal to 0.5 is taken

* Coverage cutoff of 11 (50% of average coverage) was applied to all the RNAseq

gene id	annotation	n-fold	coverage	coverage mock
AT1G02360	Chitinase family protein	6.641833471	18.93865033	2.799883
AT1G02400	gibberellin 2-oxidase 6	8.108629296	18.13839333	2.197563
AT1G02920	glutathione S-transferase 7	5.031160828	210.9697723	41.20788633
AT1G02930	glutathione S-transferase 6	3.443248601	334.9555763	95.595978
AT1G03220	Eukaryotic aspartyl protease family protein	2.515849485	215.620219	84.17407
AT1G03230	Eukaryotic aspartyl protease family protein	2.048179634	36.714883	17.59652433
AT1G03370	C2 calcium/lipid-binding and GRAM domain containing protein	2.197439659	22.657416	10.12144667
AT1G03740	Protein kinase superfamily protein	2.547676916	47.02310933	18.13293
AT1G05575	transmembrane protein	3.988301047	15.79061	3.894366
AT1G05675	UDP-Glycosyltransferase superfamily protein	28.3702643	32.77528433	1.135856333
AT1G07000	exocyst subunit exo70 family protein B2	3.695908291	31.113187	8.258087333
AT1G07128	NA	3.426881575	12.617916	3.628747333
AT1G07135	glycine-rich protein	5.432688259	76.60095567	13.84434667
AT1G07520	GRAS family transcription factor	4.90651016	16.48658533	3.310237667
AT1G08930	Major facilitator superfamily protein	2.444005767	294.6339873	118.4109087
AT1G08940	Phosphoglycerate mutase family protein	2.01508013	16.1739	7.871882667
AT1G09932	Phosphoglycerate mutase family protein	4.082765206	17.12783467	4.117754333
AT1G09940	Glutamyl-tRNA reductase family protein	2.255041889	45.60669467	19.84267667
AT1G09970	Leucine-rich receptor-like protein kinase family protein	2.099174796	154.209462	72.119504
AT1G11050	Protein kinase superfamily protein	2.515301194	16.569785	6.470627667
AT1G12805	nucleotide binding protein	3.082283606	24.76726267	7.854166667
AT1G13210	autoinhibited Ca2+/ATPase II	4.243813019	39.534502	9.158181
AT1G14200	RING/U-box superfamily protein	2.841233426	27.97683167	9.650261667
AT1G14370	protein kinase 2A	2.392046962	31.69035967	13.01933267
AT1G14540	Peroxidase superfamily protein	48.69006537	19.49776067	0.394993667
AT1G14870	PLANT CADMIUM RESISTANCE 2	4.528663925	138.8290807	30.09688233
AT1G15960	NRAMP metal ion transporter 6	2.042082972	13.71926933	6.583441
AT1G16110	wall associated kinase-like 6	2.633608887	27.02810467	10.066464
AT1G16130	wall associated kinase-like 2	3.257855678	25.13354	7.579370333
AT1G16670	Protein kinase superfamily protein	2.071236501	14.808564	7.020343
AT1G17420	lipoxygenase 3	2.669219337	11.52622167	4.247068
AT1G17990	FMN-linked oxidoreductases superfamily protein	3.301751966	25.61966167	7.640724667
AT1G18020	FMN-linked oxidoreductases superfamily protein	4.869660502	34.71360667	7.040203
AT1G18200	Rab GTPase-like A1I protein	2.027979833	11.56069267	5.581553
AT1G18390	Serine/Threonine kinase family catalytic domain protein	4.373501495	25.17808533	5.649842333
AT1G18570	myb domain protein 51	8.174179734	62.09374233	7.457329667
AT1G19020	CDP-diacylglycerol-glycerol-3-phosphate 3-phosphatidyltransferase	10.65739173	165.593745	15.237215
AT1G19180	jasmonate-zim-domain protein 1	2.257395937	79.35735433	34.567774
AT1G19380	sugar%2C putative (DUF1195)	2.577764289	221.382502	84.26704133
AT1G20350	translocase inner membrane subunit 17-1	2.183006235	13.348187	6.007944333
AT1G21100	O-methyltransferase family protein	2.584457873	110.3549067	41.95465733
AT1G21110	O-methyltransferase family protein	10.50559945	83.048776	7.759117333
AT1G21120	O-methyltransferase family protein	17.05187858	70.06564733	4.038663333
AT1G21130	O-methyltransferase family protein	3.543047932	160.9184203	44.604725
AT1G23710	hypothetical protein (DUF1645)	2.130758381	23.734082	10.92159833
AT1G24145	transmembrane protein	6.366798009	10.94509433	1.686159667
AT1G24150	formin homologue 4	4.037981706	31.26767433	7.596228333
AT1G25220	anthranilate synthase beta subunit 1	2.519286825	42.84038133	16.68920867
AT1G25390	Protein kinase superfamily protein	2.339192007	18.83642867	7.904179333
AT1G26380	FAD-binding Berberine family protein	66.47820672	24.57641933	0.362991333
AT1G26420	FAD-binding Berberine family protein	13.99610014	12.36468667	0.867803667
AT1G27020	plant/protein	2.688311912	76.23996233	27.804575
AT1G27695	glycine-rich protein	3.00563946	15.69363767	5.065684333
AT1G27730	salt tolerance zinc finger	4.8443849	27.32079733	5.535175333
AT1G27770	autoinhibited Ca2+-ATPase 1	2.307790933	60.337298	25.691739
AT1G28190	hypothetical protein	2.355779836	22.67218267	9.434725667
AT1G28380	MAC/Perforin domain-containing protein	2.393158796	83.492335	34.25259167
AT1G29690	MAC/Perforin domain-containing protein	2.45957978	47.287084	18.871268
AT1G30620	NAD(P)-binding Rossmann-fold superfamily protein	2.156394924	10.77067567	4.90366
AT1G30700	FAD-binding Berberine family protein	9.488768416	25.86954433	2.675139333
AT1G30730	FAD-binding Berberine family protein	2.480219693	28.072457	11.11168267
AT1G30755	elongation factor G%2C putative (DUF668)	5.069452056	23.67933	4.581932
AT1G32920	hypothetical protein	2.572862822	65.30611667	24.89329033
AT1G33590	Leucine-rich repeat (LRR) family protein	2.422090565	128.7051687	52.18152567
AT1G33600	Leucine-rich repeat (LRR) family protein	2.223148329	54.75695	24.17296133
AT1G33610	Leucine-rich repeat (LRR) family protein	2.515625917	26.985613	10.52391633
AT1G34370	C2H2 and C2HC zinc fingers superfamily protein	2.354407067	59.312518	24.731675
AT1G36622	transmembrane protein	6.76599765	22.43831267	3.254678
AT1G42990	basic region/leucine zipper motif 60	2.4162561	76.80422433	31.165447
AT1G43800	Plant stearoyl-acyl-carrier-protein desaturase family protein	2.301093147	97.90962233	41.70805267
AT1G49000	transmembrane protein	5.346385536	13.47361233	2.468963333
AT1G50740	Transmembrane proteins 14C	3.211755769	77.94558433	23.77810267
AT1G51270	vesicle-associated protein 1-4	2.08650157	47.200786	22.190422
AT1G51620	Protein kinase superfamily protein	4.365974347	22.77780533	5.110465333
AT1G51700	DOF zinc finger protein 1	2.312084461	21.709413	9.207562
AT1G51790	Leucine-rich repeat protein kinase family protein	2.599334941	27.479463	10.38866567
AT1G51800	Leucine-rich repeat protein kinase family protein	3.134230846	57.71464033	18.10657767
AT1G51820	Leucine-rich repeat protein kinase family protein	4.21206455	77.958028	18.19081933
AT1G51850	Leucine-rich repeat protein kinase family protein	3.130255594	25.59239367	8.036983
AT1G52200	PLAC8 family protein	3.575800673	105.2980733	28.887277
AT1G53430	Leucine-rich repeat transmembrane protein kinase	2.13306411	75.454594	34.731231
AT1G53440	Leucine-rich repeat transmembrane protein kinase	2.327030528	80.65004767	34.02271633
AT1G53625	hypothetical protein	26.50059296	23.73535433	0.878282667
AT1G55450	S-adenosyl-L-methionine-dependent methyltransferases superfamily protein	6.483289951	267.2976777	40.51312333
AT1G56060	cysteine-rich/transmembrane domain protein B	11.97296492	17.80167767	1.460723
AT1G56140	Leucine-rich repeat transmembrane protein kinase	3.292608745	41.168161	12.272576
AT1G56240	phloem protein 2-B13	8.781929366	35.884477	4.011199667
AT1G56250	phloem protein 2-B14	11.53982567	17.006713	1.443312333
AT1G56510	Disease resistance protein (TIR-NBS-LRR class)	2.267983046	30.68006833	13.28167433
AT1G56570	Tetratricopeptide repeat (TPR)-like superfamily protein	2.206008252	30.053566	13.468934
AT1G57990	purine permease 18	3.172791479	122.5419843	37.963492
AT1G59218	Disease resistance protein (CC-NBS-LRR class) family	#¡DIV/0!	17.25362933	0
AT1G59590	ZCF37	2.094129482	13.49930867	6.329184333
AT1G59870	ABC-2 and Plant PDR ABC-type transporter family protein	4.180142498	358.6535237	84.33762867
AT1G59910	Actin-binding FH2 (formin homology 2) family protein	2.119573679	26.693554	12.36102767
AT1G61340	F-box family protein	2.257081289	26.95348067	11.70692033

AT1G61360	S-locus lectin protein kinase family protein	4.280102027	21.063118	4.838863667
AT1G62300	WRKY family transcription factor	2.959820401	43.34080767	14.383051
AT1G63750	Disease resistance protein (TIR-NBS-LRR class) family	2.379131218	20.638821	8.510405667
AT1G63830	PLAC8 family protein	2.290353786	34.49003367	14.77933033
AT1G64610	Transducin/WD40 repeat-like superfamily protein	2.509296245	12.81359633	5.011350667
AT1G65390	phloem protein 2 A5	5.921007586	85.302219	14.181546
AT1G65510	transmembrane protein	2.490418946	42.89014633	16.87740433
AT1G65845	transmembrane protein	2.175277729	161.4536387	72.75675967
AT1G66090	Disease resistance protein (TIR-NBS class)	16.96831752	34.61670533	2.004396667
AT1G66160	CYS%2C MET%2C PRO%2C and GLY protein 1	2.094253628	62.45883433	29.27270633
AT1G66880	Protein kinase superfamily protein	3.015406854	10.195676	3.319286
AT1G67060	peptidase M50B-like protein	2.04026361	29.73926033	14.304562
AT1G67470	Protein kinase superfamily protein	2.260082137	12.87464067	5.588652667
AT1G68690	Protein kinase superfamily protein	3.36294718	12.45692833	3.632134333
AT1G69840	SPFH/Band 7/PHB domain-containing membrane-associated protein family	2.267336543	112.7195003	48.780341
AT1G69890	actin cross-linking protein (DUF569)	2.027286278	25.35709833	12.276702
AT1G69900	Actin cross-linking protein	2.563085333	21.66221433	8.298039
AT1G70140	formin 8	2.921848075	16.629615	5.587494333
AT1G71400	receptor like protein 12	2.966195044	16.244344	5.380382333
AT1G71697	choline kinase 1	2.093080551	42.369302	19.88480333
AT1G72060	serine-type endopeptidase inhibitor	2.401667518	82.53911067	33.71406567
AT1G72070	Chaperone DnaJ-domain superfamily protein	2.183367391	106.9060157	47.954266
AT1G72416	Chaperone DnaJ-domain superfamily protein	2.641343902	24.320368	9.035209
AT1G72520	PLAT/LH2 domain-containing lipoxygenase family protein	9.90717454	25.399827	2.520195
AT1G72900	Toll-Interleukin-Resistance (TIR) domain-containing protein	4.695195861	60.875604	12.724396
AT1G72920	Toll-Interleukin-Resistance (TIR) domain family protein	3.99454579	11.15817633	2.738565
AT1G72930	toll/interleukin-1 receptor-like protein	2.350084644	78.565779	32.85144733
AT1G72940	Toll-Interleukin-Resistance (TIR) domain-containing protein	3.055002023	33.22259833	10.664146
AT1G74330	Protein kinase superfamily protein	2.191731566	20.28789033	9.086042333
AT1G74590	glutathione S-transferase TAU 10	4.7208084	16.77970667	3.481100333
AT1G74650	myb domain protein 31	3.622422704	17.54065067	4.74997
AT1G75860	DNA ligase	2.516463597	55.334607	21.54308967
AT1G76070	hypothetical protein	2.645404164	23.13875333	8.582926667
AT1G76680	12-oxophytodienoate reductase 1	2.120855453	38.01199767	17.60759933
AT1G76970	Target of Myb protein 1	3.686093111	16.16998433	4.305965333
AT1G77500	DUF630 family protein%2C putative (DUF630 and DUF632)	2.023505178	16.04559533	7.780949333
AT1G77890	DNA-directed RNA polymerase II protein	2.124864964	15.95173567	7.358175333
AT1G78130	Major facilitator superfamily protein	3.331831463	10.22096067	3.001213333
AT1G79850	ribosomal protein S17	2.092125457	235.3521117	110.5915527
AT1G80820	cinnamoyl coa reductase	5.353761477	14.454928	2.652533667
AT1G80840	WRKY DNA-binding protein 40	3.536619078	38.97323733	10.831064
AT2G01180	phosphatidic acid phosphatase 1	2.893413067	26.86797367	9.117955667
AT2G01755	hypothetical protein	#¡DIV/0!	31.954328	0
AT2G02860	sucrose transporter 2	2.209654872	34.313469	15.24259333
AT2G04040	MATE efflux family protein	2.266776234	14.16735967	6.139199667
AT2G13790	somatic embryogenesis receptor-like kinase 4	3.411971473	49.55894967	14.24619333
AT2G13800	somatic embryogenesis receptor-like kinase 5	3.69970852	37.050801	9.824674333
AT2G14247	Expressed protein	3.200458006	30.65143067	9.407678333
AT2G15390	fucosyltransferase 4	5.119370462	22.57083333	4.33395
AT2G16060	hemoglobin 1	2.00718617	136.9669673	66.98820633
AT2G17740	Cysteine/Histidine-rich C1 domain family protein	6.860010087	15.24897733	2.182562333
AT2G17850	Rhodanese/Cell cycle control phosphatase superfamily protein	3.0165115	33.10713167	10.776324
AT2G18210	hypothetical protein	7.678182439	10.266389	1.315643333
AT2G18690	transmembrane protein	4.145345302	51.58173	12.22982033
AT2G20142	Toll-Interleukin-Resistance (TIR) domain family protein	7.438116082	41.44347367	5.461072333
AT2G20560	DNAJ heat shock family protein	2.313388941	18.36942833	7.793065333
AT2G20562	taximin	5.338151758	18.40301533	3.380135
AT2G20724	NA	2.043839169	30.94905667	14.936856
AT2G20960	phospholipase-like protein (PEARLI 4) family protein	2.995776155	89.668602	29.35520167
AT2G22500	uncoupling protein 5	4.699006772	16.64243367	3.479377333
AT2G22870	P-loop containing nucleoside triphosphate hydrolases superfamily protein	2.855664406	34.88570267	12.00404233
AT2G23320	WRKY DNA-binding protein 15	2.653096139	162.920303	60.233903
AT2G23340	DREB and EAR motif protein 3	2.059148613	10.961858	5.218352333
AT2G23680	Cold acclimation protein WCOR413 family	4.431787835	15.41319433	3.415507667
AT2G23810	tetraspanin8	2.175917208	239.758901	108.1667913
AT2G24600	Ankyrin repeat family protein	7.246505978	79.99613167	10.87421267
AT2G25735	hypothetical protein	5.155058611	45.69149767	8.699683
AT2G26010	plant defensin 1.3	2.114508989	23.955914	11.12930067
AT2G26190	calmodulin-binding family protein	3.008608692	70.87707333	23.11529633
AT2G26530	AR781%2C pheromone receptor-like protein (DUF1645)	3.887877646	70.474537	17.79337467
AT2G26560	phospholipase A 2A	19.67885113	42.54785267	2.127337667
AT2G27660	Cysteine/Histidine-rich C1 domain family protein	4.466582997	16.07576933	3.53888
AT2G28400	senescence regulator (Protein of unknown function%2C DUF584)	3.086299171	24.77310867	7.867227
AT2G28570	hypothetical protein	2.159422243	46.18624367	20.96874033
AT2G29720	FAD/NAD(P)-binding oxidoreductase family protein	2.00248688	11.17127267	5.480293
AT2G30870	glutathione S-transferase PHI 10	2.074466383	389.0453593	184.1222027
AT2G31865	poly(ADP-ribose) glycohydrolase 2	4.517075231	11.959572	2.597948333
AT2G31880	Leucine-rich repeat protein kinase family protein	4.471770008	136.9728113	30.07574167
AT2G31945	transmembrane protein	2.326493307	21.47378967	9.049619667
AT2G32020	Acyl-CoA N-acyltransferases (NAT) superfamily protein	4.952129418	15.70982433	3.113333333
AT2G32030	Acyl-CoA N-acyltransferases (NAT) superfamily protein	5.658927845	15.46525233	2.685732
AT2G32180	plastid transcriptionally active 18	2.052169521	40.93650833	19.69880933
AT2G32200	cysteine-rich/transmembrane domain A-like protein	2.196319165	10.416086	4.655738
AT2G32210	cysteine-rich/transmembrane domain A-like protein	2.168993262	25.090629	11.339169
AT2G32240	early endosome antigen	2.204817591	71.36810567	31.81002
AT2G32690	glycine-rich protein 23	2.171846035	36.38999233	16.52744833
AT2G34410	O-acetyltransferase family protein	6.121344689	32.63256533	5.253064
AT2G35710	Nucleotide-diphospho-sugar transferases superfamily protein	3.551533055	15.26830633	4.222243667
AT2G35930	plant U-box 23	4.976368154	64.619746	12.74682333
AT2G36220	hypothetical protein	3.010391985	37.08805033	12.079847
AT2G37940	Inositol phosphorylceramide synthase 2	4.056187872	62.100285	15.0316
AT2G38470	WRKY DNA-binding protein 33	6.069916326	150.9202833	24.397437
AT2G38870	Serine protease inhibitor%2C potato inhibitor I-type family protein	3.10242243	337.761851	106.7905327
AT2G39200	Seven transmembrane MLO family protein	7.103204142	111.0734227	15.37534233
AT2G39210	Major facilitator superfamily protein	2.382405247	76.79427367	31.64944333
AT2G39420	alpha/beta-Hydrolases superfamily protein	2.164631445	16.127889	7.321235
AT2G39518	Uncharacterized protein family (UPF0497)	2.98170363	31.14316	10.267586
AT2G40140	zinc finger (CCCH-type) family protein	3.166963098	61.21367633	18.96522833
AT2G40830	RING-H2 finger C1A	2.755223856	22.04435867	7.876376667
AT2G41100	Calcium-binding EF hand family protein	2.490290422	775.4604907	305.692554
AT2G44370	Cysteine/Histidine-rich C1 domain family protein	8.786357563	40.27099333	4.501850333
AT2G44380	Cysteine/Histidine-rich C1 domain family protein	2.135690092	20.543796	9.436175667
AT2G44490	Glycosyl hydrolase superfamily protein	3.390051284	273.0904287	79.11665867
AT2G46430	cyclic nucleotide gated channel 3	6.911451847	15.674764	2.230020667

AT2G46750	D-arabinono-1%2C4-lactone oxidase family protein	2.030512261	11.770862	5.695038
AT2G47060	Protein kinase superfamily protein	2.1449551	52.897608	24.196924
AT2G48121	ribonuclease III family protein	3.446126802	33.93410433	9.555342667
AT3G02550	LOB domain-containing protein 41	2.027443753	125.9280523	60.914196
AT3G04640	glycine-rich protein	2.355652033	20.85171833	8.678235
AT3G07195	RPM1-interacting protein 4 (RIN4) family protein	9.730142687	19.740251	1.993970667
AT3G08710	thioredoxin H-type 9	2.219416577	42.34357033	18.71618367
AT3G08720	serine/threonine protein kinase 2	3.64601192	32.44334833	8.739925
AT3G08760	Protein kinase superfamily protein	2.235563925	17.587378	7.720581
AT3G09440	Heat shock protein 70 (Hsp 70) family protein	2.223026553	79.048106	34.913581
AT3G09830	Protein kinase superfamily protein	3.815793512	28.041063	7.216199333
AT3G09940	monodehydroascorbate reductase	3.13698765	57.359593	17.98023933
AT3G10720	Plant invertase/pectin methylesterase inhibitor superfamily	2.013927253	87.10384967	42.65786733
AT3G11820	syntaxin of plants 121	3.0909317	87.47969267	27.785613
AT3G11840	E3 ubiquitin-protein ligase PUB24-like protein	5.033950356	12.21103867	2.380371667
AT3G12580	heat shock protein 70	2.118707211	55.40965667	25.67301567
AT3G13435	transmembrane protein	3.089927166	20.84168233	6.611626667
AT3G13437	transmembrane protein	2.853545397	13.79018833	4.736191333
AT3G14200	Chaperone DnaJ-domain superfamily protein	3.344447494	23.488657	6.889304333
AT3G14620	cytochrome P450%2C family 72%2C subfamily A%2C polypeptide 8	2.036130358	33.042074	15.92799433
AT3G15356	Legume lectin family protein	5.205815739	173.413994	32.729947
AT3G15518	hypothetical protein	9.78323531	20.649244	2.066501667
AT3G16030	lectin protein kinase family protein	4.85812819	19.900509	4.015465333
AT3G16530	Legume lectin family protein	7.480836179	160.4228057	21.07202667
AT3G16720	TOXICOS EN LEVADURA 2	2.377669698	17.13396867	7.065185
AT3G17700	cyclic nucleotide-binding transporter 1	2.739648153	10.06642933	3.603016
AT3G18250	Putative membrane lipoprotein	6.43843431	12.394106	1.8901
AT3G18690	MAP kinase substrate 1	2.211110289	25.70101233	11.389846
AT3G19010	2-oxoglutarate (2OG) and Fe(II)-dependent oxygenase superfamily protein	2.664359515	160.572238	59.18017833
AT3G19660	hypothetical protein	2.251394228	13.08644133	5.694131333
AT3G21070	NAD kinase 1	3.149162007	53.641926	16.73053033
AT3G21630	chitin elicitor receptor kinase 1	2.174884723	34.83682	15.723802
AT3G23170	hypothetical protein	2.15030701	25.319898	11.54958433
AT3G23550	MATE efflux family protein	2.392453323	19.862257	8.164203333
AT3G24420	alpha/beta-Hydrolases superfamily protein	2.507600685	22.421861	8.782665333
AT3G24500	multiprotein bridging factor 1C	2.191153576	35.81398767	16.01251533
AT3G25610	ATPase E1-E2 type family protein / haloacid dehalogenase-like hydrolase family protein	4.770036689	11.710729	2.414141333
AT3G25900	Homocysteine S-methyltransferase family protein	2.149204641	25.24215833	11.51686833
AT3G26430	GDSL-like Lipase/Acylhydrolase superfamily protein	2.56232162	20.808233	7.992519333
AT3G26500	plant intracellular ras group-related LRR 2	3.995399606	17.33495067	4.257082333
AT3G26680	DNA repair metallo-beta-lactamase family protein	2.012903281	24.357851	11.890152
AT3G26910	hydroxyproline-rich glycoprotein family protein	3.218510718	30.452376	9.288448333
AT3G26980	membrane-anchored ubiquitin-fold protein 4 precursor	2.645615346	47.08431767	17.44637367
AT3G27220	Galactose oxidase/kelch repeat superfamily protein	2.297730527	43.148005	18.42652867
AT3G28210	zinc finger (AN1-like) family protein	2.621608218	13.49285267	5.053315333
AT3G29000	Calcium-binding EF-hand family protein	15.34244	10.50171	0.673128
AT3G43190	sucrose synthase 4	4.786160564	13.518113	2.773278333
AT3G44400	Disease resistance protein (TIR-NBS-LRR class) family	2.282848281	11.70761567	5.041611333
AT3G45640	mitogen-activated protein kinase 3	3.296000981	189.7872213	56.56458267
AT3G46090	C2H2 and C2HC zinc fingers superfamily protein	4.247831536	21.869724	5.053506333
AT3G46280	kinase-like protein	2.198920491	109.6680657	49.00738267
AT3G47570	Leucine-rich repeat protein kinase family protein	2.157610744	16.31943667	7.425267333
AT3G49530	NAC domain containing protein 62	2.103559202	34.55425067	16.12614367
AT3G50260	cooperatively regulated by ethylene and jasmonate 1	5.058898212	13.66804233	2.653594667
AT3G50470	homolog of RPW8 3	2.376999591	15.370652	6.341750667
AT3G50480	homolog of RPW8 4	2.219222686	361.8442577	159.9674783
AT3G50900	hypothetical protein	2.018242534	11.44207133	5.566990333
AT3G50930	cytochrome BC1 synthesi	5.406380625	18.56613967	3.375753
AT3G50950	HOPZ-ACTIVATED RESISTANCE 1	2.442400539	107.3235703	43.13438333
AT3G51920	calmodulin 9	2.010546895	218.911728	106.8396633
AT3G52400	syntaxin of plants 122	5.352020448	98.28895067	18.03597333
AT3G52430	alpha/beta-Hydrolases superfamily protein	3.008712978	18.78728733	6.12136
AT3G52450	plant U-box 22	2.953085986	15.994111	5.314559
AT3G52535	NA	2.091542527	11.683965	5.476337667
AT3G52748	NA	4.513953454	12.277243	2.671254
AT3G54150	S-adenosyl-L-methionine-dependent methyltransferases superfamily protein	16.30775622	27.97776	1.688344
AT3G55840	Hs1pro-1 protein	6.236869829	21.63993867	3.400918667
AT3G55980	salt-inducible zinc finger 1	3.69860869	99.92515	26.53191467
AT3G57450	hypothetical protein	2.76530309	77.86049633	27.595848
AT3G59080	Eukaryotic aspartyl protease family protein	2.05268867	27.164087	12.98540267
AT3G59310	solute carrier family 35 protein (DUF914)	2.03391266	17.81089867	8.609494333
AT3G59700	lectin-receptor kinase	2.248271054	11.74223133	5.132952333
AT3G60415	phosphoglycerate mutase family protein	2.427695087	13.05222133	5.276005333
AT4G00970	cysteine-rich RLK (RECEPTOR-like protein kinase) 41	2.783390564	13.58410733	4.800266333
AT4G01010	cyclic nucleotide-gated channel 13	3.453955805	19.51051733	5.541681667
AT4G01250	WRKY family transcription factor	2.467802598	98.381096	39.085972
AT4G01700	Chitinase family protein	2.007626498	23.088922	11.29468567
AT4G01750	rhamnogalacturonan xylosyltransferase 2	4.265399194	31.000641	7.135173333
AT4G02330	Plant invertase/pectin methylesterase inhibitor superfamily	2.57626042	43.523426	16.59571967
AT4G02380	senescence-associated gene 21	2.215667509	1020.639643	451.656376
AT4G03510	RING membrane-anchor 1	2.73295859	39.83349433	14.35997367
AT4G06746	related to AP2 9	2.835022569	10.97448467	3.800458
AT4G08850	Leucine-rich repeat receptor-like protein kinase family protein	2.60594739	149.9759873	56.54575733
AT4G10265	Wound-responsive family protein	2.210424858	39.48282867	17.53434233
AT4G10270	Wound-responsive family protein	2.747259743	74.65374133	26.63340167
AT4G11280	1-aminocyclopropane-1-carboxylic acid (acc) synthase 6	3.553897805	10.82433633	2.99323
AT4G11660	winged-helix DNA-binding transcription factor family protein	2.374034395	12.846918	5.299390333
AT4G11850	phospholipase D gamma 1	2.705869144	33.318278	12.088343
AT4G11890	Protein kinase superfamily protein	15.40783268	20.30410733	1.295905667
AT4G12720	MutT/nudix family protein	3.905138836	184.785491	46.39860133
AT4G13330	S-adenosyl-L-methionine-dependent methyltransferases superfamily protein	2.27162614	15.16299867	6.553469667
AT4G14220	RING-H2 group F1A	2.026080846	20.829672	10.090186
AT4G14365	hypothetical protein	9.458104609	68.95782867	7.157717333
AT4G14370	Disease resistance protein (TIR-NBS-LRR class) family	6.000656573	12.93212167	2.116747667
AT4G14890	2Fe-2S ferredoxin-like superfamily protein	#¡DIV/0!	42.36337267	0
AT4G15233	ABC-2 and Plant PDR ABC-type transporter family protein	2.792500424	20.19262133	7.107373667
AT4G16957	SUPPRESSOR OF%2C CONSTITUTIVE protein	3.330228778	17.50320567	5.149495667
AT4G17490	ethylene responsive element binding factor 6	5.314322368	65.12730433	12.01644267
AT4G18170	WRKY DNA-binding protein 28	2.084997059	28.95021833	13.64900133
AT4G18197	purine permease 7	5.002540182	29.68031233	5.829323
AT4G18205	Nucleotide-sugar transporter family protein	5.937163592	70.91398233	11.73747767
AT4G18880	heat shock transcription factor A4A	2.152989985	14.296265	6.512285
AT4G19520	disease resistance protein (TIR-NBS-LRR class) family	3.579885116	26.56477067	7.290244333
AT4G20000	VQ motif-containing protein	11.15742447	11.73814167	1.032813333

AT4G20780	calmodulin like 42	5.667508783	37.424738	6.485898667
AT4G20830	FAD-binding Berberine family protein	3.513083261	137.9975273	38.569402
AT4G20860	FAD-binding Berberine family protein	2.107724045	187.2963203	87.295667
AT4G21390	S-locus lectin protein kinase family protein	5.921291491	12.10949833	2.013255333
AT4G21830	methionine sulfoxide reductase B7	2.169646466	24.468677	11.09361633
AT4G22212	defensin-like protein	2.068971749	112.6139197	53.40421933
AT4G22470	protease inhibitor/seed storage/lipid transfer protein (LTP) family protein	3.087645561	107.2882867	34.11014933
AT4G22690	cytochrome P450%2C family 706%2C subfamily A%2C polypeptide 1	3.84855083	220.530029	56.312711
AT4G22710	cytochrome P450%2C family 706%2C subfamily A%2C polypeptide 2	4.246367618	126.327942	29.230729
AT4G23010	UDP-galactose transporter 2	2.101420385	61.260876	28.61155267
AT4G23180	cysteine-rich RLK (RECEPTOR-like protein kinase) 10	4.33209055	51.369416	11.65139167
AT4G23190	cysteine-rich RLK (RECEPTOR-like protein kinase) 11	6.154268155	78.67147667	12.55382033
AT4G23215	NA	10.73556465	17.17174067	1.570742
AT4G23220	cysteine-rich RECEPTOR-like kinase	9.478962863	10.291312	1.064177333
AT4G23270	cysteine-rich RLK (RECEPTOR-like protein kinase) 19	2.118484875	47.37607033	21.95836067
AT4G23550	WRKY family transcription factor	3.443097005	13.001104	3.70435
AT4G23570	phosphatase-like protein	2.543710655	96.29842833	37.122631
AT4G23810	WRKY family transcription factor	4.203416149	21.84115733	5.086418
AT4G24110	NADP-specific glutamate dehydrogenase	2.876296593	63.82930233	21.75940967
AT4G24160	alpha/beta-Hydrolases superfamily protein	2.306821054	41.35165867	17.60372167
AT4G24570	dicarboxylate carrier 2	3.444818359	36.31739367	10.361577
AT4G25390	Protein kinase superfamily protein	2.276054132	11.976416	5.163241333
AT4G25810	xyloglucan endotransglycosylase 6	2.129821974	344.9368893	158.969411
AT4G26200	1-amino-cyclopropane-1-carboxylate synthase 7	4.578429868	40.07627267	8.600463
AT4G27280	Calcium-binding EF-hand family protein	3.621748735	72.047226	19.51366967
AT4G27970	SLAC1 homologue 2	2.993175175	11.431009	3.748332333
AT4G28085	transmembrane protein	2.85961492	15.00291367	5.147137
AT4G29780	nuclease	2.507756045	13.11823667	5.134402333
AT4G30210	P450 reductase 2	3.111563514	282.8560123	89.27293267
AT4G30280	xyloglucan endotransglucosylase/hydrolase 18	3.950060853	496.6867577	123.4133073
AT4G31550	WRKY DNA-binding protein 11	2.879520472	110.769881	37.726963
AT4G31800	WRKY DNA-binding protein 18	2.7758017	153.7885093	54.34759133
AT4G33050	calmodulin-binding family protein	3.316697364	93.319983	27.61424133
AT4G33060	Cyclophilin-like peptidyl-prolyl cis-trans isomerase family protein	2.179315846	38.49664533	17.31914867
AT4G33070	Thiamine pyrophosphate dependent pyruvate decarboxylase family protein	2.814785529	44.547367	15.53834133
AT4G33300	ADR1-like 1	2.041360535	63.93744133	30.73630533
AT4G33560	Wound-responsive family protein	2.419589906	59.760784	24.21419033
AT4G34150	Calcium-dependent lipid-binding (CaLB domain) family protein	2.698822858	159.5127717	58.01739733
AT4G34180	Cyclase family protein	2.133348861	166.213109	76.416232
AT4G34390	extra-large GTP-binding protein 2	2.549939745	33.862644	13.040146
AT4G35480	RING-H2 finger A3B	3.492262033	11.31077633	3.176378333
AT4G36500	hypothetical protein	4.948176938	114.0044837	22.60486733
AT4G36990	heat shock factor 4	2.191772379	34.71415233	15.53243667
AT4G37370	cytochrome P450%2C family 81%2C subfamily D%2C polypeptide 8	8.680775813	20.23817467	2.287525667
AT4G37450	arabinogalactan protein 18	2.651721701	140.484492	51.956724
AT4G38540	FAD/NAD(P)-binding oxidoreductase family protein	2.265268808	88.27195	38.22824467
AT4G38550	phospholipase-like protein (PEARLI 4) family protein	5.12750288	164.557287	31.482405
AT4G39570	Galactose oxidase/kelch repeat superfamily protein	2.186117472	11.53131033	5.174306333
AT4G39640	gamma-glutamyl transpeptidase 1	2.449765114	45.29511133	18.15715033
AT4G39890	RAB GTPase homolog H1C	3.190023571	13.80036967	4.240875333
AT5G01100	O-fucosyltransferase family protein	6.614915982	39.43415333	5.853400667
AT5G01540	lectin receptor kinase a4.1	10.66684739	13.769046	1.279871667
AT5G01542	NA	5.346210554	18.65243833	3.424007333
AT5G01950	Leucine-rich repeat protein kinase family protein	2.13807569	16.05301667	7.370772
AT5G02290	Protein kinase superfamily protein	2.139191591	56.81191367	26.05996867
AT5G03380	Heavy metal transport/detoxification superfamily protein	2.008213406	109.2601923	53.43560167
AT5G04340	6	3.274801411	16.723371	5.014814333
AT5G06320	NDR1/HIN1-like 3	2.300842068	137.7595977	58.76772333
AT5G08790	NAC (No Apical Meristem) domain transcriptional regulator superfamily protein	2.044077434	61.97162367	29.753627
AT5G10040	transmembrane protein	4.789138321	26.310771	5.386206667
AT5G10380	RING/U-box superfamily protein	2.173481828	15.80378833	7.130053667
AT5G10695	methionyl-tRNA synthetase	2.603247606	38.971891	14.65402567
AT5G12340	DUF4228 domain protein	5.984916785	11.60231267	1.908496667
AT5G12880	proline-rich family protein	2.39848517	10.92376667	4.472346667
AT5G13080	WRKY DNA-binding protein 75	2.393618006	16.20041167	6.643621333
AT5G13190	GSH-induced LITAF domain protein	2.413083373	47.96995467	19.500702
AT5G14700	NAD(P)-binding Rossmann-fold superfamily protein	3.0846557	14.89314933	4.739193
AT5G15120	2-aminoethanethiol dioxygenase%2C putative (DUF1637)	2.514479362	34.32429333	13.39823867
AT5G15130	WRKY DNA-binding protein 72	5.781525174	16.982369	2.882586
AT5G15870	glycosyl hydrolase family 81 protein	2.095109931	23.75277533	11.13413367
AT5G18470	Curculin-like (mannose-binding) lectin family protein	2.030129094	14.74069333	7.123235333
AT5G19240	Glycoprotein membrane precursor GPI-anchored	7.703061989	88.483475	11.286875
AT5G20230	blue-copper-binding protein	2.070197914	265.1552073	125.5752207
AT5G22520	hypothetical protein	19.46613852	13.07358667	0.657407333
AT5G22690	Disease resistance protein (TIR-NBS-LRR class) family	3.055696204	29.63268433	9.517342667
AT5G23510	hypothetical protein	4.612138763	49.48649867	10.53003
AT5G24110	WRKY DNA-binding protein 30	35.07441543	17.97467733	0.503359
AT5G25250	SPFH/Band 7/PHB domain-containing membrane-associated protein family	8.005385666	31.44619733	3.863397333
AT5G25440	Protein kinase superfamily protein	2.154687346	46.558774	21.174698
AT5G25930	kinase family with leucine-rich repeat domain-containing protein	4.634118164	28.07625633	5.950465667
AT5G26030	ferrochelatase 1	2.074081172	45.34488633	21.48482467
AT5G26920	Cam-binding protein 60-like G	5.846490099	13.53259667	2.274085333
AT5G27420	carbon/nitrogen insensitive 1	10.17604719	29.33884233	2.830389333
AT5G28450	Chlorophyll A-B binding family protein	#¡DIV/0!	103.3852743	0
AT5G28610	LOW protein: ATP-dependent RNA helicase DRS1-like protein	9.16608585	11.79192667	1.264669333
AT5G28630	glycine-rich protein	7.222280035	65.569214	8.901682
AT5G35735	Auxin-responsive family protein	5.073301476	61.73422367	11.95165933
AT5G36925	hypothetical protein	9.507453641	22.62457333	2.340917667
AT5G38210	Protein kinase family protein	2.780317207	10.12524567	3.569154667
AT5G38430	Ribulose bisphosphate carboxylase (small chain) family protein	2.156555426	1913.949015	874.658732
AT5G39580	Peroxidase superfamily protein	2.638286901	361.6562483	134.6459837
AT5G39670	Calcium-binding EF-hand family protein	11.56521429	48.05541767	4.075740667
AT5G39890	2-aminoethanethiol dioxygenase%2C putative (DUF1637)	3.911226943	27.66084067	6.933738667
AT5G40170	receptor like protein 54	2.336297577	25.84644933	10.865102
AT5G40540	Protein kinase superfamily protein	2.159157284	21.01089833	9.542368333
AT5G40590	Cysteine/Histidine-rich C1 domain family protein	9.440084977	18.54879133	1.932557667
AT5G41100	hydroxyproline-rich glycoprotein family protein	2.920769279	23.32299267	7.840134
AT5G41180	leucine-rich repeat transmembrane protein kinase family protein	2.408348425	13.401421	5.461731
AT5G41740	Disease resistance protein (TIR-NBS-LRR class) family	4.649986542	28.63201867	6.040433667
AT5G41750	Disease resistance protein (TIR-NBS-LRR class) family	12.50240522	21.09923633	1.657281333
AT5G42830	HXXXD-type acyl-transferase family protein	3.193073558	10.90287733	3.356314667
AT5G43420	RING/U-box superfamily protein	5.909059761	13.19262867	2.191407
AT5G43520	Cysteine/Histidine-rich C1 domain family protein	5.029332309	23.100762	4.512202333
AT5G44070	phytochelatin synthase 1 (PCS1)	5.07065758	138.1705917	26.76391067

AT5G44568	transmembrane protein	2.812309219	20.96492833	7.313370667
AT5G44585	hypothetical protein	8.959403259	18.058576	1.979181
AT5G44910	Toll-Interleukin-Resistance (TIR) domain family protein	3.387072711	14.49015633	4.202359667
AT5G45340	cytochrome P450%2C family 707%2C subfamily A%2C polypeptide 3	6.407456335	22.52709933	3.452290667
AT5G46470	disease resistance protein (TIR-NBS-LRR class) family	2.076143237	31.94223433	15.10566567
AT5G46510	Disease resistance protein (TIR-NBS-LRR class) family	2.168083341	14.02389767	6.349065667
AT5G46910	Transcription factor jumonji (jmj) family protein / zinc finger (C5HC2 type) family protein	2.633147285	10.30257467	3.839779333
AT5G47230	ethylene responsive element binding factor 5	3.603787783	15.82309233	4.304916
AT5G47910	respiratory burst oxidase homologue D	2.991149541	70.68452433	23.205754
AT5G47960	RAB GTPase homolog A4C	3.620111493	11.84980167	3.214482333
AT5G48380	BAK1-interacting receptor-like kinase 1	2.822132421	52.190689	18.152176
AT5G48430	Eukaryotic aspartyl protease family protein	5.728301942	16.682312	2.862016333
AT5G48530	hypothetical protein	5.974742443	43.51456567	7.145930333
AT5G48540	receptor-like protein kinase-related family protein	6.460635294	78.29090633	11.907697
AT5G48657	defense protein-like protein	3.628813524	13.38454133	3.62484
AT5G49520	WRKY DNA-binding protein 48	2.403207665	21.646017	8.843667333
AT5G51190	Integrase-type DNA-binding superfamily protein	5.375376989	44.85975867	8.192746
AT5G52750	Heavy metal transport/detoxification superfamily protein	5.863669221	45.936209	7.695833333
AT5G54710	Ankyrin repeat family protein	5.833925276	99.14282667	16.68688167
AT5G54720	Ankyrin repeat family protein	3.035615637	16.173935	5.242342
AT5G54860	Major facilitator superfamily protein	2.556415054	15.241471	5.853104333
AT5G57220	cytochrome P450%2C family 81%2C subfamily F%2C polypeptide 2	18.56991182	77.556184	4.101047
AT5G58120	Disease resistance protein (TIR-NBS-LRR class) family	2.653866631	18.71957033	6.928793
AT5G58430	exocyst subunit exo70 family protein B1	2.024272209	69.02174	33.46516167
AT5G58940	calmodulin-binding receptor-like cytoplasmic kinase 1	3.313977249	10.67352467	3.159877667
AT5G61010	exocyst subunit exo70 family protein E2	2.27365934	22.86632033	9.875347
AT5G61560	U-box domain-containing protein kinase family protein	2.289848548	14.345476	6.150075
AT5G61910	DCD (Development and Cell Death) domain protein	2.151613292	27.10937733	12.37705667
AT5G62020	heat shock transcription factor B2A	2.288931805	15.519384	6.646332
AT5G62070	IQ-domain 23	2.167361055	28.60243167	12.935765
AT5G62520	similar to RCD one 5	3.063747722	26.973633	8.637762667
AT5G64120	Peroxidase superfamily protein	2.761265865	163.9505667	58.30327833
AT5G64310	arabinogalactan protein 1	2.967300869	89.58668033	29.630373
AT5G64660	CYS%2C MET%2C PRO%2C and GLY protein 2	2.438487123	12.719618	5.117265333
AT5G64905	elicitor peptide 3 precursor	29.61635507	32.00706467	1.059088
AT5G66070	RING/U-box superfamily protein	2.397435963	17.97778467	7.353545667
AT5G66210	calcium-dependent protein kinase 28	4.338050273	88.46566633	20.02318067
AT5G66675	transmembrane protein%2C putative (DUF677)	2.279051154	27.38989833	11.79278433
AT5G66985	hypothetical protein	3.537575257	16.83805667	4.666216667
AT5G67420	LOB domain-containing protein 37	2.045537421	20.050642	9.652203333
AT5G67490	succinate dehydrogenase assembly factor	2.14291726	51.857442	23.64299633
ATCG00010	NA	2.200346748	46.38356	20.59360733
ATCG00700	photosystem II reaction center protein N	2.350894291	10.10353533	4.212121333
AT1G02640	beta-xylosidase 2	0.488181701	25.40133567	51.05818067
AT1G04940	translocon at the inner envelope membrane of chloroplasts 20	0.387207982	14.03931167	35.54574
AT1G05250	Peroxidase superfamily protein	0.37386332	6.808311333	17.922475
AT1G09467	NA	0.440356806	7.740196	17.318627
AT1G19050	response regulator 7	0.332003717	30.717215	90.94379167
AT1G21460	Nodulin MtN3 family protein	0.469045362	12.38087433	25.935956
AT1G43790	tracheary element differentiation-related 6	0.420491646	7.177948333	16.80374633
AT1G49660	carboxyesterase 5	0.33931371	19.283565	55.79310233
AT1G55335	transmembrane protein	0	0	76.66482667
AT1G55980	FAD/NAD(P)-binding oxidoreductase family protein	0.40329352	7.060857	17.19912767
AT1G56580	plant/protein (Protein of unknown function%2C DUF538)	0	0	76.465716
AT1G60740	Thioredoxin superfamily protein	0	0	12.770842
AT1G61740	Sulfite exporter TauE/SafE family protein	0.424001796	61.92645633	143.607305
AT1G67900	Phototropic-responsive NPH3 family protein	0.372762243	4.707286667	12.41178933
AT1G70090	glucosyl transferase family 8	0.404963909	21.06832833	51.13523967
AT1G71920	HISTIDINE BIOSYNTHESIS 6B	0.275401229	2.950558333	10.55982467
AT1G75830	low-molecular-weight cysteine-rich 67	0.413001455	6.091483333	14.5378
AT1G76880	Duplicated homeodomain-like superfamily protein	0.091439864	1.074739	11.57143667
AT2G17972	transmembrane protein	0.296399001	8.854292	29.51086367
AT2G18300	basic helix-loop-helix (bHLH) DNA-binding superfamily protein	0.433777842	12.217089	27.63776467
AT2G22770	basic helix-loop-helix (bHLH) DNA-binding superfamily protein	0.42195906	7.856866667	18.31227233
AT2G25900	Zinc finger C-x8-C-x5-C-x3-H type family protein	0.008185925	0.355507	42.17611733
AT2G29960	cyclophilin 5	0.385706319	20.13638967	51.26137567
AT2G34600	jasmonate-zim-domain protein 7	0.450501682	5.283515667	11.545564
AT3G02565	NA	0	0	11.37667733
AT3G13750	beta galactosidase 1	0.48222857	252.238205	513.747396
AT3G22640	cupin family protein	0.434588802	12.89098467	29.14869367
AT3G23450	transmembrane protein	0.485854232	4.976772333	10.08790733
AT3G26420	RNA-binding (RRM/RBD/RNP motifs) family protein with retrovirus zinc finger-like domain-containing protein	0.490997998	11.312071	22.583311
AT3G28220	TRAF-like family protein	0.468605827	25.43676333	53.40911233
AT3G48100	response regulator 5	0.20354763	4.793333333	23.14919533
AT3G54830	Transmembrane amino acid transporter family protein	0.443665268	11.27377133	24.986295
AT3G57040	response regulator 9	0.376741059	15.40097833	40.108101
AT4G05050	ubiquitin 11	0.390664747	100.7419463	255.327476
AT4G14360	S-adenosyl-L-methionine-dependent methyltransferases superfamily protein	0.48036049	18.106236	36.98897333
AT4G15670	Thioredoxin superfamily protein	0.421866239	4.777352	11.14261367
AT4G19170	nine-cis-epoxycarotenoid dioxygenase 4	0.450277359	18.71974067	40.84991933
AT4G26150	cytokinin-responsive gata factor 1	0.38170197	4.094165	10.543301
AT4G26260	myo-inositol oxygenase 4	0.463591178	26.60688167	56.34276833
AT4G27160	seed storage albumin 3	0.015798202	0.477430667	29.86328133
AT4G27170	seed storage albumin 4	0	0	20.63129567
AT4G28520	cruciferin 3	0.082160401	2.066889667	24.85934433
AT4G29905	hypothetical protein	0.395897569	88.37237833	218.9150493
AT4G31030	Putative membrane lipoprotein	0.155922901	2.226178667	14.16041767
AT4G34950	Major facilitator superfamily protein	0.479590012	5.247158333	10.754139
AT4G38860	SAUR-like auxin-responsive protein family	0.418062884	6.005407333	14.10591633
AT5G01215	NA	0.214510368	9.092031667	41.65593967
AT5G04530	3-ketoacyl-CoA synthase 19	0.491289854	11.066698	22.10363033
AT5G11070	hypothetical protein	0.435487005	18.869703	42.53354533
AT5G13180	NAC domain containing protein 83	0.488882133	44.82902667	90.03954033
AT5G19090	Heavy metal transport/detoxification superfamily protein	0.122540002	2.425047667	19.41337333
AT5G22090	FAF-like protein (DUF3049)	0.420276635	32.87756033	76.85440467
AT5G24530	2-oxoglutarate (2OG) and Fe(II)-dependent oxygenase superfamily protein	0.482209739	18.66035233	37.97879933
AT5G25100	Endomembrane protein 70 protein family	0.173962545	7.932778333	44.779597
AT5G44120	RmlC-like cupins superfamily protein	0.107223417	2.692784667	24.816869
AT5G44680	DNA glycosylase superfamily protein	0.414951154	8.325696	19.70939933
AT5G54370	Late embryogenesis abundant (LEA) protein-like protein	0.454377661	5.326530667	11.53968267
AT5G54740	seed storage albumin 5	0.038177643	1.384337333	35.831726
AT5G62920	response regulator 6	0.191464781	9.332007667	47.85103733
AT5G66400	Dehydrin family protein	0.2778927	3.152391667	11.171445
