# Supplementary material for: Arabinoxylan-Oligosaccharides Act as Damage Associated Molecular Patterns in Plants Regulating Disease Resistance
Source: Front Plant Sci. 2020 Aug 7;11:1210. doi: 10.3389/fpls.2020.01210 (PMC7427311; doi:10.3389/fpls.2020.01210)
Supplement: Supplementary file 2 [file DataSheet_2.pdf]

Supplementary Table S2: Gene Ontology Biological Process-terms of the 460 up-regulated genes in Arabidopsis Col-0 upon XA3XX treatment

All results were created with ClueGO v2.5.6
Organism analyzed: Arabidopsis thaliana [3702]
Identifier types used: [EnsemblGeneID EntrezGeneID
Evidence codes used: [All]
#Genes in GO_BiologicalProcess-Custom-GOA_10.03.2020_00h00 : 24662

#All unique genes in selected ontologies: 24662 (reference set for hypergeometric test)

#Genes from Cluster#1: unique uploaded ids 451 -> corresponding genes 451 with 0 (0.0%) missing -> All genes were recognized by ClueGO.
#Genes with functional annotations in all selected Ontologies from Cluster#1: 404 (89.58%) -> 47 (10.42%) are not functionally annotated in any selected Ontology!
-> To improve the % of annotated genes chose additional ontologies.

#Genes from all Clusters associated to 267 representative Terms and Pathways (after applying general selection criteria): 315 (69.84%)
#Genes from all Clusters associated to 92 representative Terms and Pathways (after p-value significance selection criteria): 280 (62.08%)

KappaScore Grouping:
Iteration: 0 with 33 groups
Iteration: 1 with 27 groups
Iteration: 2 with 25 groups
Final KappaScore groups 25
Terms not grouped 0

Merge redundant groups with >50.0% overlap
Final group size after merging: 25

#GO All Terms Specific for Cluster #1: 92

Ontology used:
GO_BiologicalProcess-Custom-GOA_10.03.2020_00h00

Evidence codes used:
All

Identifiers used:
EnsemblGeneID
EntrezGeneID
SymbolID

List of missing Genes:
Cluster #1

Statistical Test Used **Enrichment/Depletion (Two-sided hypergeometric test)**
Use pvalue cutoff **true**
pvalue cutoff **0.05**
Correction Method Used **Bonferroni step down**
Min GO Level **3**
Max GO Level **8**
Cluster #1
Number of Genes
Min Percentage

GO Fusion **false**
GO Group **true**
Kappa Score Threshold **0.4**
Over View Term **SmallestPValue**
Group By Kappa Statistics **true**
Initial Group Size **1**
Sharing Group Percentage **50**

GO ID	GO Term	Term p value	Term p value corrected with Bonferroni step	Group p value	Group p value corrected with Bonferroni step down	GO lebels	GO groups	% Associated genes	Number of genes	Associated genes found
GO:0006979	response to oxidative stress	1.13E-10	2.20E-08	1.13E-10	2.03E-09	[3]	Group00	6.262231	32	[ACS6, AT1G19020, AT1G52200, AT1G720
GO:0010150	leaf senescence	2.99E-04	0.041004024	2.99E-04	8.98E-04	[4, 5, 6, 7, 8]	Group01	6.923077	9	[ATAF2, MLO12, OPR1, PAD4, SAG21, SERK
GO:0002239	response to oomycetes	7.50E-10	1.43E-07	7.50E-10	1.28E-08	[3, 5]	Group02	14.285714	14	[ARCK1, AT1G02360, AT2G39210, AT3G16
GO:0002679	respiratory burst involved in defense response	4.26E-05	0.00642988	4.26E-05	3.83E-04	[3, 4]	Group03	60	3	[PUB22, PUB23, PUB24]
GO:0043620	regulation of DNA-templated transcription in response to stress	1.17E-04	0.017083589	1.17E-04	8.19E-04	[4, 7, 8, 9, 10]	Group04	16.666666	5	[AT-HSFB2B, HSF A4A, HSF4, HSFB2A, WRKY
GO:0046777	protein autophosphorylation	1.01E-04	0.014851897	1.01E-04	8.08E-04	[7, 8]	Group05	6.122449	12	[AT1G11050, AT1G16670, AT1G53440, AT
GO:0007165	signal transduction	8.29E-07	1.38E-04	8.29E-07	9.95E-06	[2, 3, 4]	Group06	3.1234508	63	[AT1G30755, AT1G33590, AT1G33600, AT
GO:0080167	response to karrikin	1.71E-04	0.024583393	1.71E-04	0.001024308	[3]	Group07	6.756757	10	[AT1G33590, AT3G09440, AT4G24110, AT
GO:0009061	anaerobic respiration	1.37E-07	2.42E-05	1.37E-07	1.92E-06	[5, 6]	Group08	55.555557	5	[AT1G05575, AT2G36220, AT3G27220, AT
GO:0009266	response to temperature stimulus	3.80E-05	0.005771199	3.80E-05	3.80E-04	[3]	Group09	3.9416058	27	[ARCK1, AT-HSFB2B, AT1G16670, AT3G094
GO:0009611	response to wounding	5.05E-09	9.24E-07	5.05E-09	7.57E-08	[3]	Group10	8.130081	20	[ACS6, AT1G32920, AT1G72920, AT2G388
GO:0009723	response to ethylene	1.76E-04	0.025181581	1.76E-04	8.80E-04	[4, 5]	Group11	4.901961	15	[ACS6, ASB1, AT3G15356, AT5G51190, CEJ
GO:0009753	response to jasmonic acid	1.94E-05	0.003019503	1.94E-05	2.13E-04	[4, 5]	Group12	6.735751	13	[ACS6, AT3G15356, AT4G20860, ATAF2, FB
GO:0032350	regulation of hormone metabolic process	1.93E-04	0.027250317	1.93E-04	7.73E-04	[4]	Group13	8.219178	7	[ASB1, AT3G09830, CAD1, CBP60G, NSL1,
GO:0010337	regulation of salicylic acid metabolic process	1.95E-06	3.20E-04	1.93E-04	7.73E-04	[5, 6]	Group13	20.833334	6	[AT3G09830, CAD1, CBP60G, NSL1, PAD4]
GO:1901652	response to peptide	3.41E-04	0.045981281	3.41E-04	3.41E-04	[4, 5]	Group14	33.333332	3	[AT1G30755, GSTF10, GSTF6]
GO:0043434	response to peptide hormone	2.30E-04	0.031950591	3.41E-04	3.41E-04	[4, 5, 6]	Group14	37.5	3	[AT1G30755, GSTF10, GSTF6]
GO:0006970	response to osmotic stress	3.26E-04	0.044399314	3.26E-04	6.53E-04	[3]	Group15	3.7865748	22	[ARCK1, AT2G26190, CAM9, CCR2, CRCK1,
GO:0009651	response to salt stress	2.18E-04	0.030564047	3.26E-04	6.53E-04	[4]	Group15	4.040404	20	[ARCK1, AT2G26190, CAM9, CCR2, CRCK1,
GO:0012501	programmed cell death	1.74E-06	2.88E-04	1.44E-17	3.03E-16	[3]	Group16	8.396947	12	[BCS1, CAD1, HR3, HR4, MPK3, NSL1, NUDT
GO:0034050	host programmed cell death induced by symbiont	3.64E-07	6.19E-05	1.44E-17	3.03E-16	[4]	Group16	10.752688	11	[BCS1, CAD1, HR3, HR4, MPK3, NSL1, NUDT
GO:0045087	innate immune response	5.87E-18	1.19E-15	1.44E-17	3.03E-16	[3, 4, 5, 6]	Group16	9.326425	37	[AT1G61360, AT2G32240, AT3G15356, AT
GO:0009626	plant-type hypersensitive response	3.26E-07	5.57E-05	1.44E-17	3.03E-16	[4, 5, 6, 7]	Group16	10.869565	11	[BCS1, CAD1, HR3, HR4, MPK3, NSL1, NUDT
GO:0016310	phosphorylation	3.23E-09	5.97E-07	3.23E-09	5.16E-08	[5]	Group17	3.7460318	59	[APK2A, ARCK1, AT1G03740, AT1G11050,
GO:0016301	kinase activity	2.90E-10	5.59E-08	3.23E-09	5.16E-08	[6]	Group17	4.0055246	58	[APK2A, ARCK1, AT1G11050, AT1G16670,
GO:0006468	protein phosphorylation	4.40E-11	8.66E-09	3.23E-09	5.16E-08	[6, 7]	Group17	4.553571	51	[APK2A, ARCK1, AT1G11050, AT1G16670,
GO:0004672	protein kinase activity	2.09E-11	4.17E-09	3.23E-09	5.16E-08	[7, 8]	Group17	4.6490426	51	[APK2A, ARCK1, AT1G11050, AT1G16670,
GO:0004674	protein serine/threonine kinase activity	3.18E-10	6.10E-08	3.23E-09	5.16E-08	[8, 9]	Group17	4.8098435	43	[APK2A, ARCK1, AT1G11050, AT1G16670,
GO:0010941	regulation of cell death	7.25E-08	1.29E-05	1.62E-07	2.11E-06	[4]	Group18	12.244898	12	[AT4G34180, BIR1, CEJ1, GILP, HSP70, PAD
GO:0048585	negative regulation of response to stimulus	1.71E-05	0.002684449	1.62E-07	2.11E-06	[4]	Group18	6.0240965	15	[AT5G39670, AT5G66070, BIR1, CPK28, GI
GO:0043067	regulation of programmed cell death	3.69E-04	0.049420679	1.62E-07	2.11E-06	[5]	Group18	8.641975	7	[AT4G34180, GILP, HSP70, RBOHD, RING1,
GO:0060548	negative regulation of cell death	1.54E-07	2.68E-05	1.62E-07	2.11E-06	[5]	Group18	21.052631	8	[AT4G34180, BIR1, GILP, HSP70, RBOHD, S
GO:0031348	negative regulation of defense response	2.06E-07	3.56E-05	1.62E-07	2.11E-06	[5, 6]	Group18	14.285714	10	[AT5G39670, BIR1, CPK28, GILP, HLECRK, I
GO:0043069	negative regulation of programmed cell death	9.69E-06	0.001541109	1.62E-07	2.11E-06	[6]	Group18	19.35484	6	[AT4G34180, GILP, HSP70, RBOHD, SYP121
GO:0009620	response to fungus	4.67E-11	9.16E-09	7.89E-26	1.81E-24	[3, 5]	Group19	5.736434	37	[AT2G32240, AT2G38870, AT3G13437, AT
GO:0098542	defense response to other organism	2.39E-23	4.93E-21	7.89E-26	1.81E-24	[3, 4, 5]	Group19	6.185567	73	[ADR1-L1, AT1G61360, AT2G32240, AT2G3
GO:0045087	innate immune response	5.87E-18	1.19E-15	7.89E-26	1.81E-24	[3, 4, 5, 6]	Group19	9.326425	37	[AT1G61360, AT2G32240, AT3G15356, AT
GO:0050832	defense response to fungus	5.26E-08	9.41E-06	7.89E-26	1.81E-24	[4, 5, 6]	Group19	5.2064633	29	[AT2G32240, AT2G38870, AT3G13437, AT
GO:0009814	defense response	2.46E-09	4.64E-07	7.89E-26	1.81E-24	[4, 5, 6, 7]	Group19	8.962264	19	[AT2G32240, AT3G15356, AT3G23170, CB
GO:0009816	defense response to bacterium	2.13E-05	0.003302155	7.89E-26	1.81E-24	[5, 6, 7, 8]	Group19	13.461538	7	[CBP60G, CNI1, CRK11, NHL3, PAD4, PLDG
GO:0009817	defense response to fungus	8.53E-06	0.001364954	7.89E-26	1.81E-24	[5, 6, 7, 8]	Group19	12.698413	8	[AT2G32240, AT3G15356, CERK1, GSTF7,
GO:0033554	cellular response to stress	4.71E-43	1.01E-40	5.31E-32	1.28E-30	[3]	Group20	7.819549	105	[AT-HSFB2B, AT1G02360, AT1G03220, AT1
GO:0070482	response to oxygen levels	1.85E-75	4.00E-73	5.31E-32	1.28E-30	[3]	Group20	26.9103	81	[AT-HSFB2B, AT1G02360, AT1G03220, AT1
GO:0070887	cellular response to chemical stimulus	3.90E-36	8.19E-34	5.31E-32	1.28E-30	[3]	Group20	6.3157897	109	[AT-HSFB2B, AT1G02360, AT1G03220, AT1
GO:0001666	response to hypoxia	1.62E-76	3.53E-74	5.31E-32	1.28E-30	[3, 5]	Group20	27.645052	81	[AT-HSFB2B, AT1G02360, AT1G03220, AT1
GO:0036293	response to decreased oxygen levels	1.37E-75	2.98E-73	5.31E-32	1.28E-30	[4]	Group20	27	81	[AT-HSFB2B, AT1G02360, AT1G03220, AT1
GO:0071453	cellular response to oxygen levels	1.66E-77	3.63E-75	5.31E-32	1.28E-30	[4]	Group20	29.811321	79	[AT-HSFB2B, AT1G02360, AT1G03220, AT1
GO:0036294	cellular response to decreased oxygen levels	1.66E-77	3.63E-75	5.31E-32	1.28E-30	[5]	Group20	29.811321	79	[AT-HSFB2B, AT1G02360, AT1G03220, AT1
GO:0071456	cellular response to hypoxia	5.93E-78	1.31E-75	5.31E-32	1.28E-30	[4, 6]	Group20	30.152672	79	[AT-HSFB2B, AT1G02360, AT1G03220, AT1
GO:0033037	polysaccharide localization	4.74E-07	8.01E-05	4.75E-11	9.49E-10	[3]	Group21	13.559322	9	[CAD1, CYP81F2, EXO70B1, EXO70E2, MYB
GO:0042430	indole-containing compound metabolic process	3.05E-09	5.67E-07	4.75E-11	9.49E-10	[4]	Group21	13.186813	13	[ASB1, AT3G55840, AT4G34180, CAD1, CY
GO:0052545	callose localization	2.54E-07	4.37E-05	4.75E-11	9.49E-10	[4]	Group21	14.545455	9	[CAD1, CYP81F2, EXO70B1, EXO70E2, MYB
GO:0052542	defense response by callose deposition	1.36E-08	2.46E-06	4.75E-11	9.49E-10	[4, 5]	Group21	20	9	[CAD1, CYP81F2, EXO70B1, EXO70E2, MYB
GO:0042436	indole-containing compound catabolic process	5.94E-07	9.98E-05	4.75E-11	9.49E-10	[5]	Group21	25	6	[AT3G55840, AT4G34180, CAD1, PEN2, PE
GO:0052386	cell wall thickening	7.97E-06	0.001283872	4.75E-11	9.49E-10	[5, 6]	Group21	13.333333	7	[CAD1, CYP81F2, FH4, MYB51, PEN2, PEN3]
GO:0052543	callose deposition in cell wall	5.93E-05	0.008838341	4.75E-11	9.49E-10	[4, 5, 6, 7]	Group21	11.904762	6	[CAD1, CYP81F2, MYB51, PEN2, PEN3]
GO:0052482	defense response by cell wall thickening	7.10E-07	1.19E-04	4.75E-11	9.49E-10	[4, 6, 7]	Group21	18.75	7	[CAD1, CYP81F2, FH4, MYB51, PEN2, PEN3]
GO:0052544	defense response by callose deposition in cell wall	9.69E-06	0.001541109	4.75E-11	9.49E-10	[4, 5, 6, 7, 8]	Group21	16.129032	6	[CAD1, CYP81F2, MYB51, PEN2, PEN3]
GO:0042343	indole glucosinolate metabolic process	4.10E-09	7.55E-07	4.75E-11	9.49E-10	[5, 6, 7, 8]	Group21	28	8	[CAD1, CYP81F2, IGMT1, IGMT2, MYB51, P
GO:0042344	indole glucosinolate catabolic process	3.16E-05	0.004842225	4.75E-11	9.49E-10	[6, 7, 8, 9]	Group21	25	4	[CAD1, PEN2, PEN3]
GO:0001101	response to acid chemical	3.05E-11	6.08E-09	3.61E-21	7.94E-20	[3]	Group22	4.331013	56	[ACS6, ARCK1, AT1G65510, AT1G77500, AT
GO:0010033	response to organic substance	1.44E-22	2.96E-20	3.61E-21	7.94E-20	[3]	Group22	4.7391095	99	[ACS6, ARCK1, ASB1, AT-HSFB2B, AT1G075
GO:0010035	response to inorganic substance	7.02E-05	0.010391553	3.61E-21	7.94E-20	[3]	Group22	3.2139578	36	[AR781, ARCK1, AT1G77500, AT2G32240,
GO:0042493	response to drug	3.86E-41	8.19E-39	3.61E-21	7.94E-20	[3]	Group22	12.825279	69	[AT-HSFB2B, AT1G07520, AT1G65510, AT3
GO:0046677	response to antibiotic	5.96E-09	1.08E-06	3.61E-21	7.94E-20	[3]	Group22	7.6642337	21	[AT1G65510, AT5G44568, ATAF2, BCS1, CR
GO:1901698	response to nitrogen compound	3.16E-37	6.67E-35	3.61E-21	7.94E-20	[3]	Group22	15.976332	54	[AT-HSFB2B, AT1G07520, AT1G30755, AT1
GO:1901700	response to oxygen-containing compound	9.30E-26	1.92E-23	3.61E-21	7.94E-20	[3]	Group22	5.458769	94	[ACS6, ARCK1, AT-HSFB2B, AT1G07520, AT1
GO:0009725	response to hormone	2.52E-09	4.73E-07	3.61E-21	7.94E-20	[3, 4]	Group22	3.6479445	63	[ACS6, ARCK1, ASB1, AT1G30755, AT1G336
GO:0010243	response to organonitrogen compound	6.58E-42	1.40E-39	3.61E-21	7.94E-20	[4]	Group22	20.075758	53	[AT-HSFB2B, AT1G07520, AT1G30755, AT3
GO:0014070	response to organic cyclic compound	1.47E-07	2.57E-05	3.61E-21	7.94E-20	[4]	Group22	6.3636365	21	[AT1G65510, AT5G44568, ATAF2, ATPMEP
GO:0009751	response to salicylic acid	1.31E-10	2.55E-08	3.61E-21	7.94E-20	[4, 5]	Group22	11.3924055	18	[AT1G65510, AT5G44568, ATAF2, BCS1, CR
GO:0010200	response to chitin	4.26E-48	9.16E-46	3.61E-21	7.94E-20	[4, 5]	Group22	32.63889	47	[AT-HSFB2B, AT1G07520, AT3G15356, AT3
GO:0006952	defense response	1.24E-35	2.60E-33	9.22E-41	2.31E-39	[3]	Group23	6.5313888	104	[ACS6, ADR1-L1, APK2A, ARCK1, AT1G6136
GO:0043207	response to external biotic stimulus	4.62E-32	9.61E-30	9.22E-41	2.31E-39	[3]	Group23	6.4693737	95	[ADR1-L1, ARCK1, ASB1, AT1G02360, AT1G
GO:0051707	response to other organism	4.62E-32	9.61E-30	9.22E-41	2.31E-39	[2, 4]	Group23	6.4693737	95	[ADR1-L1, ARCK1, ASB1, AT1G02360, AT1G
GO:0009617	response to bacterium	2.80E-22	5.71E-20	9.22E-41	2.31E-39	[3, 5]	Group23	8.80734	49	[ADR1-L1, ASB1, AT1G16670, AT3G09830,
GO:0009620	response to fungus	4.67E-11	9.16E-09	9.22E-41	2.31E-39	[3, 5]	Group23	5.736434	37	[AT2G32240, AT2G38870, AT3G13437, AT
GO:0034050	host programmed cell death induced by symbiont	3.64E-07	6.19E-05	9.22E-41	2.31E-39	[4]	Group23	10.752688	11	[BCS1, CAD1, HR3, HR4, MPK3, NSL1, NUDT
GO:0098542	defense response to other organism	2.39E-23	4.93E-21	9.22E-41	2.31E-39	[3, 4, 5]	Group23	6.185567	73	[ADR1-L1, AT1G61360, AT2G32240, AT2G3
GO:0045087	innate immune response	5.87E-18	1.19E-15	9.22E-41	2.31E-39	[3, 4, 5, 6]	Group23	9.326425	37	[AT1G61360, AT2G32240, AT3G15356, AT
GO:0042742	defense response to bacterium	1.92E-16	3.88E-14	9.22E-41	2.31E-39	[4, 5, 6]	Group23	8.149779	38	[ADR1-L1, AT3G09830, BIR1, CAD1, CBP60
GO:0050832	defense response to fungus	5.26E-08	9.41E-06	9.22E-41	2.31E-39	[4, 5, 6]	Group23	5.2064633	29	[AT2G32240, AT2G38870, AT3G13437, AT
GO:0009626	plant-type hypersensitive response	3.26E-07	5.57E-05	9.22E-41	2.31E-39	[4, 5, 6, 7]	Group23	10.869565	11	[BCS1, CAD1, HR3, HR4, MPK3, NSL1, NUDT
GO:0009814	defense response	2.46E-09	4.64E-07	9.22E-41	2.31E-39	[4, 5, 6, 7]	Group23	8.962264	19	[AT2G32240, AT3G15356, AT3G23170, CB
GO:0002682	regulation of immune system process	3.51E-06	5.72E-04	5.46E-11	1.04E-09	[3]	Group24	8.571428	12	[AT4G22470, AT5G25440, CBP60G, CERK1,
GO:0048583	regulation of response to stimulus	1.77E-08	3.18E-06	5.46E-11	1.04E-09	[3]	Group24	4.6134663	37	[ARCK1, AT1G56060, AT4G22470, AT5G25
GO:0002831	regulation of response to biotic stimulus	2.68E-09	5.01E-07	5.46E-11	1.04E-09	[4]	Group24	9.473684	18	[AT4G22470, AT5G25440, AT5G39670, CB
GO:0010941	regulation of cell death	7.25E-08	1.29E-05	5.46E-11	1.04E-09	[4]	Group24	12.244898	12	[AT4G34180, BIR1, CEJ1, GILP, HSP70, PAD
GO:0032101	regulation of response to external stimulus	6.55E-10	1.25E-07	5.46E-11	1.04E-09	[4]	Group24	9.693877	19	[AT4G22470, AT5G25440, AT5G39670, CB
GO:0048585	negative regulation of response to stimulus	1.71E-05	0.002684449	5.46E-11	1.04E-09	[4]	Group24	6.0240965	15	[AT5G39670, AT5G66070, BIR1, CPK28, GI
GO:0050776	regulation of immune response	4.44E-05	0.006656425	5.46E-11	1.04E-09	[4]	Group24	7.9365077	10	[AT4G22470, AT5G25440, CBP60G, CERK1,
GO:0080134	regulation of response to stress	2.41E-12	4.85E-10	5.46E-11	1.04E-09	[4]	Group24	7.4879227	31	[ARCK1, AT1G56060, AT4G22470, AT5G25
GO:0002237	response to molecule of bacterial origin	7.74E-08	1.37E-05	5.46E-11	1.04E-09	[4, 6]	Group24	22.857143	8	[AT1G16670, BCS1, CBP60G, CERK1, EXO7
GO:0002238	response to molecule of fungal origin	2.79E-04	0.038531184	5.46E-11	1.04E-09	[4, 6]	Group24	20	4	[CERK1, EXO70B2, IOS1, RING1]
GO:0002832	negative regulation of response to biotic stimulus	3.70E-04	0.049230147	5.46E-11	1.04E-09	[5]	Group24	13.157895	5	[AT5G39670, CPK28, GILP, HLECRK, IOS1]
GO:0002833	positive regulation of response to biotic stimulus	1.46E-04	0.021232882	5.46E-11	1.04E-09	[5]	Group24	8.602151	8	[AT4G22470, AT5G25440, CERK1, EXO70B
GO:0031347	regulation of defense response	3.58E-11	7.08E-09	5.46E-11	1.04E-09	[5]	Group24	8.695652	24	[ARCK1, AT4G22470, AT5G25440, AT5G39
GO:0032102	negative regulation of response to external stimulus	3.70E-04	0.049230147	5.46E-11	1.04E-09	[5]	Group24	13.157895	5	[AT5G39670, CPK28, GILP, HLECRK, IOS1]
GO:0032103	positive regulation of response to external stimulus	1.83E-04	0.025946792	5.46E-11	1.04E-09	[5]	Group24	8.333333	8	[AT4G22470, AT5G25440, CERK1, EXO70B
GO:0060548	negative regulation of cell death	1.54E-07	2.68E-05	5.46E-11	1.04E-09	[5]	Group24	21.052631	8	[AT4G34180, BIR1, GILP, HSP70, RBOHD, S
GO:0031348	negative regulation of defense response	2.06E-07	3.56E-05	5.46E-11	1.04E-09	[5, 6]	Group24	14.285714	10	[AT5G39670, BIR1, CPK28, GILP, HLECRK, I
GO:0031349	positive regulation of defense response	6.95E-06	0.001125178	5.46E-11	1.04E-09	[5, 6]	Group24	9.803922	10	[ARCK1, AT4G22470, AT5G25440, CERK1,

50, AT4G20830, AT5G39580, AT5G64120, BCB, CRCK1, CRK11, ERF6, FC1, GGT1, GSTF10, GSTF6, HEMA2, HSF A4A, LOX4, LRR XI-23, MPK3, MSRB7, NUDT7, PAP1, PCR2, PER4, PLDGAMMA1, PTI1-4, SAG21, STZ, WRKY30, WRKY53, ZAT7]
4, WRKY22, WRKY30, WRKY53]
530, AT3G23550, AT4G01700, AT4G38540, BCB, GSTF6, HLECRK, IOS1, LECRKA4.1, PAP1, WRR4]

75]
1G56140, AT5G25440, AT5G38210, CERK1, CPK28, HLECRK, LRR XI-23, WAKL2, ZAR1]
1G33610, AT1G51270, AT1G63750, AT1G66090, AT1G67470, AT1G72900, AT1G72920, AT1G72940, AT3G09830, AT3G15356, AT3G44400, AT4G08850, AT4G14370, AT5G22690, AT5G25440, AT5G25930, AT5G41750, AT5G44910, AT5G46510, AT5G51190, AT5G58120, BCS1, BZIP60, CAM9, CCR2, CEJ1, CERK1, CPK28, DEAR3, ERF5, ERF6, HLEC
4G27280, AT5G18470, AT5G48540, CYP81D8, GSTF10, GSTF6, GSTF7]
5G10040, AT5G15120]
40, AT4G34150, ATPMEPCRB, BCB, CCR2, CEJ1, CRCK1, CZF1, ERD6, ERF5, GSTF7, HSF A4A, HSF4, HSFB2A, HSP70, MBF1C, MPK3, NAC062, RBOHD, S6K2, SAG21, STZ, TCH3, WRKY33]
70, AT3G15356, ATAF2, BCB, CK1, FBS1, JAZ1, LOX3, LOX4, MPK3, NHL3, OPR1, PP2-A5, RBOHD, STZ, SUT2, WRKY40]
1, DEAR3, ERF5, ERF6, HR4, MBF1C, PAD4, PAP1, RAP2.9, SAG21, WRKY6]
S1, HR4, JAZ1, LOX3, MDHAR, PAP1, PP2-A5, SYP121, SYP122]
PAD4]

ERD6, FBS1, FUT4, GSTF10, GSTF6, MPK3, NSL1, NUDT7, PEN2, PEN3, PGN, RBOHD, RHC1A, S6K2, SRO5, STZ, WRKY33]
ERD6, FBS1, FUT4, GSTF10, GSTF6, NSL1, NUDT7, PEN2, PEN3, PGN, RHC1A, S6K2, SRO5, STZ, WRKY33]
T7, PAD4, PAP1, PLA2A, RING1]
T7, PAD4, PAP1, PLA2A]
3G23170, BCS1, CAD1, CBP60G, CCR2, CERK1, CES101, CNI1, CRK11, CYP81F2, GSTF7, HR3, HR4, MLO12, MPK3, MYB51, NAC062, NHL3, NSL1, NUDT7, PAD4, PAP1, PEN2, PEN3, PLA2A, PLDGAMMA1, PP2-A5, PROPEP3, SGT1A, WRKY11, WRKY33, WRKY53, WRR4]
T7, PAD4, PAP1, PLA2A]
AT1G16670, AT1G25390, AT1G51620, AT1G51790, AT1G51820, AT1G51850, AT1G53430, AT1G53440, AT1G56140, AT1G61360, AT1G66880, AT1G67470, AT3G09830, AT3G46280, AT3G47570, AT4G08850, AT4G25390, AT5G01950, AT5G25440, AT5G25930, AT5G38210, AT5G40540, AT5G41180, AT5G61560, ATSIK, B120, BIR1, CCR2, CERK1,
AT1G25390, AT1G51620, AT1G51790, AT1G51820, AT1G51850, AT1G53430, AT1G53440, AT1G56140, AT1G61360, AT1G66880, AT1G67470, AT3G09830, AT3G46280, AT3G47570, AT4G08850, AT4G25390, AT5G01950, AT5G25440, AT5G25930, AT5G38210, AT5G40540, AT5G41180, AT5G61560, ATSIK, B120, BIR1, CCR2, CERK1, CES101, CK1
AT1G25390, AT1G51620, AT1G51820, AT1G53440, AT1G56140, AT1G61360, AT1G66880, AT1G67470, AT3G09830, AT3G47570, AT4G08850, AT4G25390, AT5G25440, AT5G25930, AT5G38210, AT5G40540, AT5G41180, AT5G61560, ATSIK, B120, BIR1, CCR2, CERK1, CES101, CK1, CPK28, CRCK1, CRK10, CRK11, CRK14, CRK19, CRK41, FC1, HLEC
AT1G25390, AT1G51620, AT1G51820, AT1G53440, AT1G56140, AT1G61360, AT1G66880, AT1G67470, AT3G09830, AT3G47570, AT4G08850, AT4G25390, AT5G25440, AT5G25930, AT5G38210, AT5G40540, AT5G41180, AT5G61560, ATSIK, B120, BIR1, CCR2, CERK1, CES101, CK1, CPK28, CRCK1, CRK10, CRK11, CRK14, CRK19, CRK41, FC1, HLEC
AT1G25390, AT1G51820, AT1G53440, AT1G56140, AT1G61360, AT1G66880, AT1G67470, AT3G47570, AT4G08850, AT4G25390, AT5G25930, AT5G38210, AT5G61560, B120, CCR2, CERK1, CES101, CK1, CPK28, CRCK1, CRK10, CRK11, CRK14, CRK19, CRK41, FC1, HLECRK, IOS1, LECRKA4.1, MPK3, NAK, PERK9, S6K2, SERK4, SERK5, SOBIR1, WAKL2
4, RBOHD, RING1, SERK4, SOBIR1, SYP121, SYP122]
LP, GSTF10, GSTF6, HLECRK, HSP70, IOS1, PAD4, PEN3, RHC1A, SYP121, SYP122]
SYP121, SYP122]
ERK4, SYP121, SYP122]
OS1, PAD4, PEN3, SYP121, SYP122]
, SYP122]
3G15356, AT4G20830, AT4G22212, AT5G39580, AT5G64120, ATAF2, ATPMEPCRB, CERK1, CES101, CYP81F2, CZF1, EXO70B1, EXO70B2, GSTF7, HR3, HR4, IOS1, LOX3, MDHAR, MLO12, PARG2, PDF1.3, PEN2, PEN3, PGN, RBOHD, RING1, SGT1A, SYP121, SYP122, WRKY18, WRKY33, WRKY40, WRR4]
38870, AT3G09830, AT3G13437, AT3G15356, AT3G23170, AT3G23550, AT4G20830, AT4G22212, AT5G36925, AT5G39580, AT5G64120, BCS1, BIR1, CAD1, CBP60G, CCR2, CEJ1, CERK1, CES101, CNI1, CRK10, CRK11, CRK14, CRK19, CYP81F2, CZF1, EXO70B1, GSTF10, GSTF6, GSTF7, HLECRK, HR3, HR4, IOS1, JAZ1, LECRKA4.1, MLO12, MPK3, MYB51
3G23170, BCS1, CAD1, CBP60G, CCR2, CERK1, CES101, CNI1, CRK11, CYP81F2, GSTF7, HR3, HR4, MLO12, MPK3, MYB51, NAC062, NHL3, NSL1, NUDT7, PAD4, PAP1, PEN2, PEN3, PLA2A, PLDGAMMA1, PP2-A5, PROPEP3, SGT1A, WRKY11, WRKY33, WRKY53, WRR4]
3G15356, AT4G20830, AT4G22212, AT5G39580, AT5G64120, CERK1, CYP81F2, CZF1, EXO70B1, GSTF7, HR3, HR4, MLO12, PARG2, PDF1.3, PEN2, PEN3, PGN, RBOHD, SGT1A, SYP121, SYP122, WRKY18, WRKY33, WRKY40, WRR4]
P60G, CERK1, CNI1, CRK11, GSTF7, MLO12, NAC062, NHL3, PAD4, PEN2, PEN3, PLDGAMMA1, PP2-A5, WRKY33, WRKY53, WRR4]
AMMA1, WRKY53]
MLO12, PEN2, PEN3, WRR4]
G07135, AT1G14200, AT1G19020, AT1G23710, AT1G26380, AT1G50740, AT1G66090, AT1G66880, AT1G69890, AT1G72900, AT1G72920, AT1G72940, AT2G14247, AT2G25735, AT2G26190, AT2G32200, AT2G32210, AT2G36220, AT3G04640, AT3G09440, AT3G23170, AT3G27220, AT4G10265, AT4G19520, AT4G20860, AT4G24110, AT4G241
G07135, AT1G14200, AT1G19020, AT1G23710, AT1G26380, AT1G50740, AT1G66090, AT1G66880, AT1G69890, AT1G72900, AT1G72920, AT1G72940, AT2G14247, AT2G25735, AT2G26190, AT2G32200, AT2G32210, AT2G36220, AT3G04640, AT3G23170, AT3G27220, AT4G10265, AT4G19520, AT4G20860, AT4G24110, AT4G24160, AT4G272
G07135, AT1G14200, AT1G19020, AT1G23710, AT1G26380, AT1G33600, AT1G50740, AT1G66090, AT1G66880, AT1G69890, AT1G72900, AT1G72920, AT1G72940, AT1G77500, AT2G14247, AT2G25735, AT2G26190, AT2G32200, AT2G32210, AT2G36220, AT3G04640, AT3G09440, AT3G15356, AT3G23170, AT3G27220, AT4G08850, AT4G102
G07135, AT1G14200, AT1G19020, AT1G23710, AT1G26380, AT1G50740, AT1G66090, AT1G66880, AT1G69890, AT1G72900, AT1G72920, AT1G72940, AT2G14247, AT2G25735, AT2G26190, AT2G32200, AT2G32210, AT2G36220, AT3G04640, AT3G23170, AT3G27220, AT4G10265, AT4G19520, AT4G20860, AT4G24110, AT4G24160, AT4G272
G07135, AT1G14200, AT1G19020, AT1G23710, AT1G26380, AT1G50740, AT1G66090, AT1G66880, AT1G69890, AT1G72900, AT1G72920, AT1G72940, AT2G14247, AT2G25735, AT2G26190, AT2G32200, AT2G32210, AT2G36220, AT3G04640, AT3G23170, AT3G27220, AT4G10265, AT4G19520, AT4G20860, AT4G24110, AT4G24160, AT4G272
G07135, AT1G14200, AT1G19020, AT1G23710, AT1G26380, AT1G50740, AT1G66090, AT1G66880, AT1G69890, AT1G72900, AT1G72920, AT1G72940, AT2G14247, AT2G25735, AT2G26190, AT2G32200, AT2G32210, AT2G36220, AT3G04640, AT3G23170, AT3G27220, AT4G10265, AT4G19520, AT4G20860, AT4G24110, AT4G24160, AT4G272
G07135, AT1G14200, AT1G19020, AT1G23710, AT1G26380, AT1G50740, AT1G66090, AT1G66880, AT1G69890, AT1G72900, AT1G72920, AT1G72940, AT2G14247, AT2G25735, AT2G26190, AT2G32200, AT2G32210, AT2G36220, AT3G04640, AT3G23170, AT3G27220, AT4G10265, AT4G19520, AT4G20860, AT4G24110, AT4G24160, AT4G272
G07135, AT1G14200, AT1G19020, AT1G23710, AT1G26380, AT1G50740, AT1G66090, AT1G66880, AT1G69890, AT1G72900, AT1G72920, AT1G72940, AT2G14247, AT2G25735, AT2G26190, AT2G32200, AT2G32210, AT2G36220, AT3G04640, AT3G23170, AT3G27220, AT4G10265, AT4G19520, AT4G20860, AT4G24110, AT4G24160, AT4G272
51, NSL1, PEN2, PEN3]
P81F2, IGMT1, IGMT2, MPK3, MYB51, PEN2, PEN3, WRKY33]
51, NSL1, PEN2, PEN3]
51, NSL1, PEN2, PEN3]
N3]
]

]

EN2, PEN3]

T2G26190, AT2G32020, AT2G32240, AT3G13437, AT3G15356, AT4G20860, AT5G44568, ATAF2, BCS1, CAM9, CCR2, CNI1, CRCK1, CRK19, CYP707A3, ERD6, EXO70B1, EXO70B2, FBS1, GRP23, GSTF10, GSTF6, HR4, IOS1, JAZ1, LECRKA4.1, LOX3, LOX4, MBF1C, MDHAR, MPK3, NHL3, NUDT7, OPR1, PAD4, PAP1, PEN3, PGN, PMZ, PP2-A5, PUB22, PUB2
20, AT1G16670, AT1G30755, AT1G33600, AT1G65510, AT2G26190, AT2G32020, AT2G32240, AT3G09440, AT3G15356, AT3G16530, AT4G08850, AT4G20860, AT4G27280, AT5G25930, AT5G44568, AT5G46910, AT5G51190, AT5G66070, ATAF2, ATL2, ATPMEPCRB, BCS1, BZIP60, CAM9, CBP60G, CEJ1, CERK1, CMPG1, CMPG2, CNI1, CRCK1, CRK1
AT3G09440, AT3G13437, CAD1, CAM9, CCR2, CRCK1, CYP707A3, ERD6, EXO70B1, EXO70B2, GSTF10, GSTF6, GSTF7, HSP70, LOX4, MBF1C, MDHAR, MSRB7, NUDT7, OPR1, PAP1, PLA2A, PLDGAMMA1, PUB22, PUB23, SAG21, STOP1, STZ, WRKY30, WRKY33, WRKY53]
G15356, AT3G16530, AT5G44568, AT5G46910, AT5G51190, AT5G66070, ATAF2, ATL2, BCS1, BZIP60, CEJ1, CERK1, CMPG1, CMPG2, CNI1, CRCK1, CRK19, CZF1, DIC2, DOF1, EDA39, ERD6, ERF5, ERF6, FBS1, GRP23, HB1, HR4, HSF A4A, HSFB2A, IOS1, LOX4, MPK3, MSRB7, MYB31, NAC062, NHL3, NUDT7, OPR1, PAD4, PMZ, PP2-A5, PUB22, PUB23, P
CK1, CRK19, FBS1, GRP23, HR4, LOX4, NHL3, NUDT7, OPR1, PAD4, PP2-A5, SYP121, SYP122, WRKY18, WRKY30, WRKY40, WRKY53]
G77500, AT3G15356, AT3G16530, AT5G46910, AT5G51190, AT5G66070, ATL2, BZIP60, CEJ1, CERK1, CMPG1, CMPG2, CNI1, CZF1, DOF1, EDA39, ERD6, ERF5, ERF6, FBS1, GSTF10, GSTF6, HSF A4A, HSFB2A, IOS1, MPK3, MYB31, NAC062, PEN3, PMZ, PUB22, PUB23, PUB24, RHA3B, RING1, RMA1, SERK4, STZ, SZF1, WRKY11, WRKY15, WRKY18, WRKY
1G30755, AT1G65510, AT1G77500, AT2G26190, AT2G32020, AT2G32240, AT3G13437, AT3G15356, AT3G16530, AT4G20860, AT5G44568, AT5G46910, AT5G51190, AT5G66070, ATAF2, ATL2, ATPMEPCRB, BCS1, BZIP60, CAM9, CCR2, CEJ1, CERK1, CMPG1, CMPG2, CNI1, CRCK1, CRK19, CYP707A3, CZF1, DOF1, EDA39, ERD6, ERF5, ERF6, EXO70B1
500, AT1G65510, AT2G26190, AT2G32020, AT2G32240, AT3G15356, AT4G08850, AT4G20860, AT4G27280, AT5G25930, AT5G44568, AT5G51190, ATAF2, ATPMEPCRB, BCS1, CAM9, CEJ1, CNI1, CRCK1, CRK19, DEAR3, ERD6, ERF5, ERF6, FBS1, GRP23, GSTF10, GSTF6, HB1, HR4, IOS1, JAZ1, LECRKA4.1, LOX3, MBF1C, MDHAR, MPK3, MYB31, NHL3, C
G15356, AT3G16530, AT5G46910, AT5G51190, AT5G66070, ATL2, BZIP60, CEJ1, CERK1, CMPG1, CMPG2, CNI1, CZF1, DOF1, EDA39, ERD6, ERF5, ERF6, FBS1, GSTF10, GSTF6, HSF A4A, HSFB2A, IOS1, MPK3, MYB31, NAC062, PEN3, PMZ, PUB22, PUB23, PUB24, RHA3B, RING1, RMA1, SERK4, STZ, SZF1, WRKY11, WRKY15, WRKY18, WRKY22, WRKY29,
CRB, BCS1, CRK19, FBS1, GRP23, HR4, NHL3, OPR1, PAD4, PEN3, PP2-A5, SERK4, SYP121, SYP122, WRKY18, WRKY30, WRKY40, WRKY53]
K19, FBS1, GRP23, HR4, NHL3, OPR1, PAD4, PP2-A5, SYP121, SYP122, WRKY18, WRKY30, WRKY40, WRKY53]
G16530, AT5G46910, AT5G51190, AT5G66070, ATL2, BZIP60, CEJ1, CERK1, CMPG1, CMPG2, CNI1, CZF1, DOF1, EDA39, ERD6, ERF5, ERF6, HSF A4A, HSFB2A, IOS1, MPK3, MYB31, NAC062, PMZ, PUB22, PUB23, PUB24, RHA3B, RING1, SERK4, STZ, SZF1, WRKY11, WRKY15, WRKY18, WRKY22, WRKY29, WRKY33, WRKY40, WRKY48, WRKY53, WRKY6, Z
0, AT1G66090, AT2G32240, AT2G38870, AT3G09830, AT3G13437, AT3G15356, AT3G23170, AT3G23550, AT3G55840, AT4G20830, AT4G22212, AT4G34180, AT5G36925, AT5G39580, AT5G44568, AT5G64120, AT5G66070, ATL2, AtIPCS2, BCS1, BIR1, CAD1, CBP60G, CCR2, CEJ1, CERK1, CES101, CNI1, CPK28, CRK10, CRK11, CRK14, CRK19, CYP8
16670, AT1G61360, AT2G32240, AT2G38870, AT2G39210, AT3G09440, AT3G09830, AT3G13437, AT3G15356, AT3G16530, AT3G23170, AT3G23550, AT4G01700, AT4G20830, AT4G22212, AT4G38540, AT5G36925, AT5G39580, AT5G64120, ATAF2, ATPMEPCRB, BCB, BCS1, BIR1, CAD1, CBP60G, CCR2, CEJ1, CERK1, CES101, CNBT1, CNI1, CRK10,
16670, AT1G61360, AT2G32240, AT2G38870, AT2G39210, AT3G09440, AT3G09830, AT3G13437, AT3G15356, AT3G16530, AT3G23170, AT3G23550, AT4G01700, AT4G20830, AT4G22212, AT4G38540, AT5G36925, AT5G39580, AT5G64120, ATAF2, ATPMEPCRB, BCB, BCS1, BIR1, CAD1, CBP60G, CCR2, CEJ1, CERK1, CES101, CNBT1, CNI1, CRK10,
BCS1, BIR1, CAD1, CBP60G, CEJ1, CERK1, CNI1, CRK10, CRK11, CRK14, CRK19, CYP81F2, EXO70B1, EXO70B2, FBS1, GSTF10, GSTF6, GSTF7, HLECRK, HR4, HSP70, IOS1, JAZ1, LECRKA4.1, LOX4, MPK3, MYB51, NHL3, NUDT7, PAD4, PEN2, PEN3, PLDGAMMA1, RING1, RPS6, TET8, WRKY11, WRKY18, WRKY33, WRKY40, WRKY48, WRKY53, XLG2, ZAR1]
3G15356, AT4G20830, AT4G22212, AT5G39580, AT5G64120, ATAF2, ATPMEPCRB, CERK1, CES101, CYP81F2, CZF1, EXO70B1, EXO70B2, GSTF7, HR3, HR4, IOS1, LOX3, MDHAR, MLO12, PARG2, PDF1.3, PEN2, PEN3, PGN, RBOHD, RING1, SGT1A, SYP121, SYP122, WRKY18, WRKY33, WRKY40, WRR4]
T7, PAD4, PAP1, PLA2A]
38870, AT3G09830, AT3G13437, AT3G15356, AT3G23170, AT3G23550, AT4G20830, AT4G22212, AT5G36925, AT5G39580, AT5G64120, BCS1, BIR1, CAD1, CBP60G, CCR2, CEJ1, CERK1, CES101, CNI1, CRK10, CRK11, CRK14, CRK19, CYP81F2, CZF1, EXO70B1, GSTF10, GSTF6, GSTF7, HLECRK, HR3, HR4, IOS1, JAZ1, LECRKA4.1, MLO12, MPK3, MYB51
3G23170, BCS1, CAD1, CBP60G, CCR2, CERK1, CES101, CNI1, CRK11, CYP81F2, GSTF7, HR3, HR4, MLO12, MPK3, MYB51, NAC062, NHL3, NSL1, NUDT7, PAD4, PAP1, PEN2, PEN3, PLA2A, PLDGAMMA1, PP2-A5, PROPEP3, SGT1A, WRKY11, WRKY33, WRKY53, WRR4]
G, CEJ1, CERK1, CNI1, CRK10, CRK11, CRK14, CRK19, CYP81F2, EXO70B1, GSTF10, GSTF6, GSTF7, HLECRK, JAZ1, LECRKA4.1, MYB51, NHL3, NUDT7, PAD4, PEN2, PEN3, PLDGAMMA1, RPS6, TET8, WRKY11, WRKY18, WRKY33, WRKY40, WRKY48, WRKY53, XLG2, ZAR1]
3G15356, AT4G20830, AT4G22212, AT5G39580, AT5G64120, CERK1, CYP81F2, CZF1, EXO70B1, GSTF7, HR3, HR4, MLO12, PARG2, PDF1.3, PEN2, PEN3, PGN, RBOHD, SGT1A, SYP121, SYP122, WRKY18, WRKY33, WRKY40, WRR4]
T7, PAD4, PAP1, PLA2A]
P60G, CERK1, CNI1, CRK11, GSTF7, MLO12, NAC062, NHL3, PAD4, PEN2, PEN3, PLDGAMMA1, PP2-A5, WRKY33, WRKY53, WRR4]
, GILP, MKS1, NUDT7, SGT1A, SYP121, SYP122, WRKY18, WRKY40]
440, AT5G39670, AT5G66070, ATAF2, BIR1, CBP60G, CERK1, CPK28, EXO70B1, EXO70B2, GILP, GSTF10, GSTF6, HLECRK, HSP70, IOS1, JAZ1, MKS1, MPK3, NAC062, NUDT7, PAD4, PARG2, PEN3, RHC1A, SGT1A, SOBIR1, SYP121, SYP122, WRKY11, WRKY18, WRKY40, WRKY53, WRKY75]
P60G, CERK1, CPK28, EXO70B2, GILP, HLECRK, IOS1, MKS1, NUDT7, PAD4, SGT1A, SYP121, SYP122, WRKY18, WRKY40]
4, RBOHD, RING1, SERK4, SOBIR1, SYP121, SYP122]
P60G, CERK1, CPK28, EXO70B2, GILP, HLECRK, IOS1, MKS1, NUDT7, PAD4, SGT1A, SYP121, SYP122, WRKY18, WRKY40, WRKY75]
LP, GSTF10, GSTF6, HLECRK, HSP70, IOS1, PAD4, PEN3, RHC1A, SYP121, SYP122]
, GILP, MKS1, NUDT7, SGT1A, SYP121, SYP122]
440, AT5G39670, BIR1, CBP60G, CERK1, CPK28, EXO70B2, GILP, GSTF10, GSTF6, HLECRK, HSP70, IOS1, JAZ1, MKS1, MPK3, NAC062, NUDT7, PAD4, PARG2, PEN3, SGT1A, SOBIR1, SYP121, SYP122, WRKY18, WRKY40, WRKY53]
0B2, IOS1, WRKY18, WRKY40]

2, IOS1, MKS1, NUDT7, PAD4]
670, BIR1, CBP60G, CERK1, CPK28, EXO70B2, GILP, HLECRK, IOS1, JAZ1, MKS1, NUDT7, PAD4, PEN3, SGT1A, SOBIR1, SYP121, SYP122, WRKY18, WRKY40, WRKY53]

2, IOS1, MKS1, NUDT7, PAD4]
ERK4, SYP121, SYP122]
OS1, PAD4, PEN3, SYP121, SYP122]
EXO70B2, IOS1, MKS1, NUDT7, PAD4, SOBIR1]

RK, IOS1, JAZ1, LECRKA4.1, MBF1C, MKS1, MPK3, NUDT7, PAD4, PAP1, PERK9, PP2-A5, RABA4C, RABA6b, RABH1c, RAP2.9, RBOHD, RLP12, RLP54, SERK4, SERK5, SYP121, SYP122, TIR, WAKL2, WAKL6, WRKY6, WRR4, XLG2]

CES101, CK1, CPK28, CRCK1, CRK10, CRK11, CRK14, CRK19, CRK41, FC1, HLECRK, IOS1, LECRKA4.1, LRR XI-23, MPK3, NADK1, NAK, PERK9, RLP54, S6K2, SERK4, SERK5, SOBIR1, WAKL2, WAKL6, ZAR1]
, CPK28, CRCK1, CRK10, CRK11, CRK14, CRK19, CRK41, FC1, HLECRK, IOS1, LECRKA4.1, LRR XI-23, MPK3, NADK1, NAK, PERK9, RLP54, S6K2, SERK4, SERK5, SOBIR1, WAKL2, WAKL6, ZAR1]
RK, IOS1, LECRKA4.1, LRR XI-23, MPK3, NAK, PERK9, S6K2, SERK4, SERK5, SOBIR1, WAKL2, WAKL6, ZAR1]
RK, IOS1, LECRKA4.1, LRR XI-23, MPK3, NAK, PERK9, S6K2, SERK4, SERK5, SOBIR1, WAKL2, WAKL6, ZAR1]
, WAKL6, ZAR1]

, NAC062, NHL3, NSL1, NUDT7, PAD4, PAP1, PARG2, PDF1.3, PEN2, PEN3, PGN, PLA2A, PLDGAMMA1, PP2-A5, PROPEP3, RBOHD, RPS6, SGT1A, SYP121, SYP122, TET8, WRKY11, WRKY18, WRKY33, WRKY40, WRKY48, WRKY53, WRR4, XLG2, ZAR1]

60, AT4G27280, AT4G29780, AT4G33070, AT4G33560, AT4G34150, AT5G03380, AT5G15120, AT5G19240, AT5G35735, AT5G39890, AT5G58120, AT5G66985, BCB, BCS1, BZIP60, CAD1, CBP60G, CNI1, CYP707A3, CYP81F2, CZF1, DIC2, FBS1, FH4, FLOT1, FTM1, GILP, GSTF7, HB1, HR3, HR4, HSF A4A, HSF4, HSFB2A, HSP70, LBD41, MBF1C, MPK3, M
80, AT4G29780, AT4G33070, AT4G33560, AT4G34150, AT5G03380, AT5G15120, AT5G19240, AT5G35735, AT5G39890, AT5G58120, AT5G66985, BCB, CBP60G, CNI1, CYP707A3, CYP81F2, CZF1, DIC2, FBS1, FLOT1, FTM1, GGT1, GILP, GSTF7, HB1, HSP70, LBD41, MBF1C, NHL3, NUDT7, OPR1, PAD4, PAP1, PER4, PERK9, PLA2A, PROPEP3, RBOHD, S
65, AT4G19520, AT4G20860, AT4G24110, AT4G24160, AT4G27280, AT4G29780, AT4G33070, AT4G33560, AT4G34150, AT5G03380, AT5G15120, AT5G19240, AT5G25930, AT5G35735, AT5G39890, AT5G51190, AT5G58120, AT5G64120, AT5G66985, BCB, BCS1, BZIP60, CAD1, CBP60G, CEJ1, CERK1, CNI1, CYP707A3, CYP81F2, CZF1, DEAR3, DIC
80, AT4G29780, AT4G33070, AT4G33560, AT4G34150, AT5G03380, AT5G15120, AT5G19240, AT5G35735, AT5G39890, AT5G58120, AT5G66985, BCB, CBP60G, CNI1, CYP707A3, CYP81F2, CZF1, DIC2, FBS1, FLOT1, FTM1, GGT1, GILP, GSTF7, HB1, HSP70, LBD41, MBF1C, NHL3, NUDT7, OPR1, PAD4, PAP1, PER4, PERK9, PLA2A, PROPEP3, RBOHD, S
80, AT4G29780, AT4G33070, AT4G33560, AT4G34150, AT5G03380, AT5G15120, AT5G19240, AT5G35735, AT5G39890, AT5G58120, AT5G66985, BCB, CBP60G, CNI1, CYP707A3, CYP81F2, CZF1, DIC2, FBS1, FLOT1, FTM1, GGT1, GILP, GSTF7, HB1, HSP70, LBD41, MBF1C, NHL3, NUDT7, OPR1, PAD4, PAP1, PER4, PERK9, PLA2A, PROPEP3, RBOHD, S
80, AT4G29780, AT4G33070, AT4G33560, AT4G34150, AT5G03380, AT5G15120, AT5G19240, AT5G35735, AT5G39890, AT5G58120, AT5G66985, BCB, CBP60G, CNI1, CYP707A3, CYP81F2, CZF1, DIC2, FBS1, FLOT1, FTM1, GILP, GSTF7, HB1, HSP70, LBD41, MBF1C, NHL3, NUDT7, OPR1, PAP1, PER4, PERK9, PLA2A, PROPEP3, RBOHD, S6K2, SERK4, S
80, AT4G29780, AT4G33070, AT4G33560, AT4G34150, AT5G03380, AT5G15120, AT5G19240, AT5G35735, AT5G39890, AT5G58120, AT5G66985, BCB, CBP60G, CNI1, CYP707A3, CYP81F2, CZF1, DIC2, FBS1, FLOT1, FTM1, GILP, GSTF7, HB1, HSP70, LBD41, MBF1C, NHL3, NUDT7, OPR1, PAP1, PER4, PERK9, PLA2A, PROPEP3, RBOHD, S6K2, SERK4, S
80, AT4G29780, AT4G33070, AT4G33560, AT4G34150, AT5G03380, AT5G15120, AT5G19240, AT5G35735, AT5G39890, AT5G58120, AT5G66985, BCB, CBP60G, CNI1, CYP707A3, CYP81F2, CZF1, DIC2, FBS1, FLOT1, FTM1, GILP, GSTF7, HB1, HSP70, LBD41, MBF1C, NHL3, NUDT7, OPR1, PAP1, PER4, PERK9, PLA2A, PROPEP3, RBOHD, S6K2, SERK4, S

23, SAG21, STZ, SYP121, SYP122, WRKY18, WRKY30, WRKY33, WRKY40, WRKY53, XLG2]
9, CZF1, DEAR3, DOF1, EDA39, ERD6, ERF5, ERF6, EXO70B2, FBS1, GRP23, GSTF10, GSTF6, HB1, HR4, HSF A4A, HSFB2A, HSP70, IOS1, JAZ1, LECRKA4.1, LOX3, MBF1C, MDHAR, MPK3, MYB31, NAC062, NHL3, OPR1, PAD4, PAP1, PEN3, PGN, PMZ, PP2-A5, PUB22, PUB23, PUB24, RAP2.9, RHA3B, RING1, RMA1, SAG21, SERK4, SGT1A, STZ, SYP121, SYP12

PUB24, RHA3B, RING1, SERK4, STZ, SYP121, SYP122, SZF1, UCP5, WRKY11, WRKY15, WRKY18, WRKY22, WRKY29, WRKY30, WRKY33, WRKY40, WRKY48, WRKY53, WRKY6, ZAT6, ZAT7]

22, WRKY29, WRKY33, WRKY40, WRKY48, WRKY53, WRKY6, ZAT6, ZAT7]
, EXO70B2, FBS1, GRP23, GSTF10, GSTF6, HB1, HR4, HSF A4A, HSFB2A, IOS1, JAZ1, LECRKA4.1, LOX3, LOX4, MBF1C, MDHAR, MPK3, MSRB7, MYB31, NAC062, NHL3, NUDT7, OPR1, PAD4, PAP1, PEN3, PGN, PMZ, PP2-A5, PUB22, PUB23, PUB24, RHA3B, RING1, SAG21, SERK4, STZ, SYP121, SYP122, SZF1, WRKY11, WRKY15, WRKY18, WRKY22, WRKY2
OPR1, PAD4, PAP1, PEN3, PGN, PMZ, PP2-A5, RAP2.9, SAG21, SERK4, SGT1A, STZ, SYP121, SYP122, WRKY18, WRKY30, WRKY40, WRKY53, WRKY6, XLG2]
WRKY33, WRKY40, WRKY48, WRKY53, WRKY6, ZAT6, ZAT7]

ZAT6, ZAT7]
1F2, CZF1, ERF5, ERF6, EXO70B1, EXO70B2, EXO70E2, FH4, GSTF10, GSTF6, GSTF7, HLECRK, HR3, HR4, IOS1, JAZ1, LECRKA4.1, LOX3, LOX4, MKS1, MLO12, MPK3, MYB51, NAC062, NAK, NHL3, NSL1, NUDT7, PAD4, PAP1, PARG2, PDF1.3, PEN2, PEN3, PGN, PLA2A, PLDGAMMA1, PP2-A5, PROPEP3, PUB22, PUB23, PUB24, RBOHD, RHA3B, RING1, RPS
, CRK11, CRK14, CRK19, CYP81F2, CZF1, ERF6, EXO70B1, EXO70B2, FBS1, GSTF10, GSTF6, GSTF7, HLECRK, HR3, HR4, HSP70, IOS1, JAZ1, LECRKA4.1, LOX3, LOX4, MDHAR, MLO12, MPK3, MYB51, NAC062, NHL3, NSL1, NUDT7, PAD4, PAP1, PARG2, PDF1.3, PEN2, PEN3, PGN, PLA2A, PLDGAMMA1, PP2-A5, PROPEP3, RBOHD, RING1, RPS6, SAG21, SGT
, CRK11, CRK14, CRK19, CYP81F2, CZF1, ERF6, EXO70B1, EXO70B2, FBS1, GSTF10, GSTF6, GSTF7, HLECRK, HR3, HR4, HSP70, IOS1, JAZ1, LECRKA4.1, LOX3, LOX4, MDHAR, MLO12, MPK3, MYB51, NAC062, NHL3, NSL1, NUDT7, PAD4, PAP1, PARG2, PDF1.3, PEN2, PEN3, PGN, PLA2A, PLDGAMMA1, PP2-A5, PROPEP3, RBOHD, RING1, RPS6, SAG21, SGT

, NAC062, NHL3, NSL1, NUDT7, PAD4, PAP1, PARG2, PDF1.3, PEN2, PEN3, PGN, PLA2A, PLDGAMMA1, PP2-A5, PROPEP3, RBOHD, RPS6, SGT1A, SYP121, SYP122, TET8, WRKY11, WRKY18, WRKY33, WRKY40, WRKY48, WRKY53, WRR4, XLG2, ZAR1]

MYB51, NAC062, NHL3, NSL1, NUDT7, OPR1, PAD4, PAP1, PARG2, PEN2, PEN3, PER4, PERK9, PLA2A, PROPEP3, RBOHD, RMA1, S6K2, SAG21, SERK4, SGT1A, SNM1, SRO5, STZ, SUS4, SZF1, TCH3, UCP5, WRKY22, WRKY33, WRKY6, WRKY75, XTH18, ZAT6, ZAT7, pEARLI4]
6K2, SERK4, SGT1A, SRO5, STZ, SUS4, SZF1, TCH3, UCP5, WRKY22, XTH18, ZAT6, ZAT7]
2, ERF5, ERF6, FBS1, FLOT1, FTM1, GILP, GSTF7, HB1, HSP70, IOS1, JAZ1, LBD41, LECRKA4.1, MBF1C, MPK3, NHL3, NUDT7, OPR1, PAD4, PAP1, PEN3, PER4, PERK9, PLA2A, PP2-A5, PROPEP3, RAP2.9, RBOHD, RMA1, S6K2, SAG21, SERK4, SGT1A, SRO5, STZ, SUS4, SYP121, SYP122, SZF1, TCH3, UCP5, WRKY22, WRKY6, XTH18, ZAT6, ZAT7]
6K2, SERK4, SGT1A, SRO5, STZ, SUS4, SZF1, TCH3, UCP5, WRKY22, XTH18, ZAT6, ZAT7]
6K2, SERK4, SGT1A, SRO5, STZ, SUS4, SZF1, TCH3, UCP5, WRKY22, XTH18, ZAT6, ZAT7]
GT1A, SRO5, STZ, SUS4, SZF1, TCH3, UCP5, WRKY22, XTH18, ZAT6, ZAT7]
GT1A, SRO5, STZ, SUS4, SZF1, TCH3, UCP5, WRKY22, XTH18, ZAT6, ZAT7]
GT1A, SRO5, STZ, SUS4, SZF1, TCH3, UCP5, WRKY22, XTH18, ZAT6, ZAT7]

22, SZF1, WRKY11, WRKY15, WRKY18, WRKY22, WRKY29, WRKY30, WRKY33, WRKY40, WRKY48, WRKY53, WRKY6, XLG2, ZAT6, ZAT7]

9, WRKY30, WRKY33, WRKY40, WRKY48, WRKY53, WRKY6, XLG2, ZAT6, ZAT7]

6, SAG21, SGT1A, SOBIR1, SYP121, SYP122, TET8, TIR, WRKY11, WRKY18, WRKY22, WRKY29, WRKY33, WRKY40, WRKY48, WRKY53, WRKY72, WRR4, XLG2, ZAR1]
1A, SYP121, SYP122, TET8, WRKY11, WRKY18, WRKY33, WRKY40, WRKY48, WRKY53, WRR4, XLG2, ZAR1]
1A, SYP121, SYP122, TET8, WRKY11, WRKY18, WRKY33, WRKY40, WRKY48, WRKY53, WRR4, XLG2, ZAR1]
