# Supplementary material for: Arabinoxylan-Oligosaccharides Act as Damage Associated Molecular Patterns in Plants Regulating Disease Resistance
Source: Front Plant Sci. 2020 Aug 7;11:1210. doi: 10.3389/fpls.2020.01210 (PMC7427311; doi:10.3389/fpls.2020.01210)
Supplement: Supplementary file 3 [file DataSheet_3.pdf]

Supplementary Table S3: carbohydrates used in this work .

Acronym	Name	Source
XA3XX	33-α-L-arabinofuranosyl-xylotetraose	Megazyme (O-XA3XX)
XA2XX	23-α-L-arabinofuranosyl-xylotetraose	Megazyme (O-XA2XX)
A23XX	23,33-di-α-L-arabinofuranosyl-xylotriose	Megazyme (O-A23XX)
A2XX	23-α-L-arabinofuranosyl-xylotriose	Megazyme (O-A2XX)
Chi6, Chitohexaose	β-1,4-D-(GlcNAc)6	Megazyme (O-CHI6)
Arabinoxylan (rye)	Rye arabinoxylan	Megazyme (P-RAXY)
Xylan (birch)	Xylan from birch wood	Sigma-Aldrich (95588)
Xylan (beech)	Xylan from beech wood	Sigma-Aldrich (X4252)
Xylan (oat)	Xylan from oat spelts	Sigma-Aldrich (95590)
Arabinan (sugar beech)	Debranched arabinan from sugar beet	Megazyme (P-DBAR)
Arabinoxylan (wheat)	Low viscosity wheat flour arabinoxylan	Megazyme (P-WAXYL)
