# Supplementary material for: Arabinoxylan-Oligosaccharides Act as Damage Associated Molecular Patterns in Plants Regulating Disease Resistance
Source: Front Plant Sci. 2020 Aug 7;11:1210. doi: 10.3389/fpls.2020.01210 (PMC7427311; doi:10.3389/fpls.2020.01210)
Supplement: Supplementary file 4 [file DataSheet_4.pdf]

Supplementary Table S4: Oligonucleotides used in this work.

Gene	AGI locus	Forward oligonucleotide	Reverse oligonucleotide
UBQ21	*AT5G25760*	GCTCTTATCAAAGGACCTTCGG	CGAACTTGAGGAGGTTGCAAAG
CYP81F2	*AT5G57220*	TATTGTCCGCATGGTCACAGG	CCACTGTTGTCATTGATGTCCG
WRKY53	*AT4G23810*	CACCAGAGTCAAACCAGCCATTA	CTTTACCATCATCAAGCCCATCGG
FRK1	*AT2G19190*	ATCTTCGCTTGGAGCTTCTC	TGCAGCGCAAGGACTAGAG
PHI1	*AT1G35140*	TTGGTTTAGACGGGATGGTG	ACTCCAGTACAAGCCGATCC
NHL10	*AT2G35980*	TTCCTGTCCGTAACCCAAAC	CCCTCGTAGTAGGCATGAGC
