# Supplementary figures and images for: Arabinoxylan-Oligosaccharides Act as Damage Associated Molecular Patterns in Plants Regulating Disease Resistance
Source: Front Plant Sci. 2020 Aug 7;11:1210. doi: 10.3389/fpls.2020.01210 (PMC7427311; doi:10.3389/fpls.2020.01210)

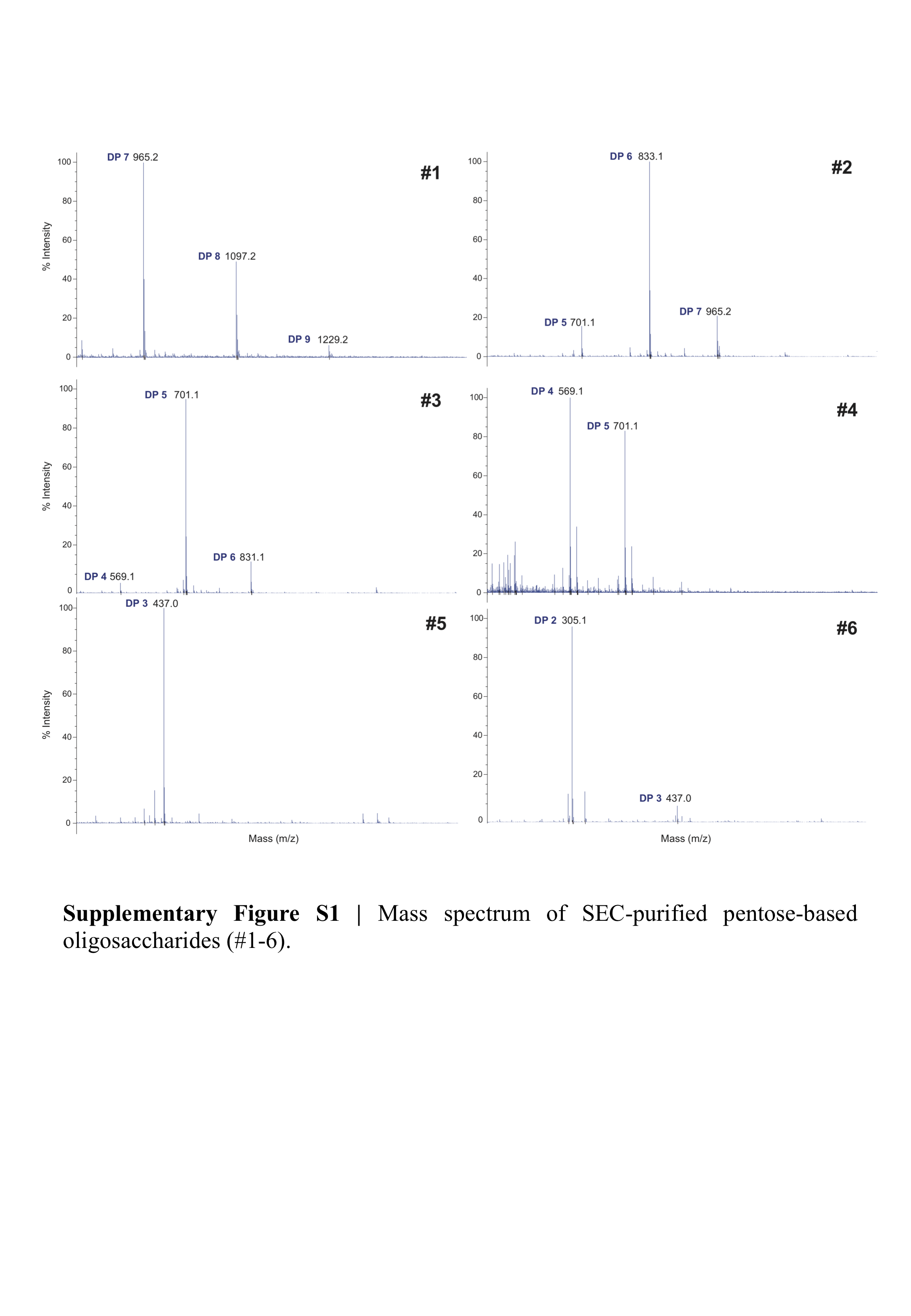

Supplement: Supplementary file 5 [file Image_1.tiff]

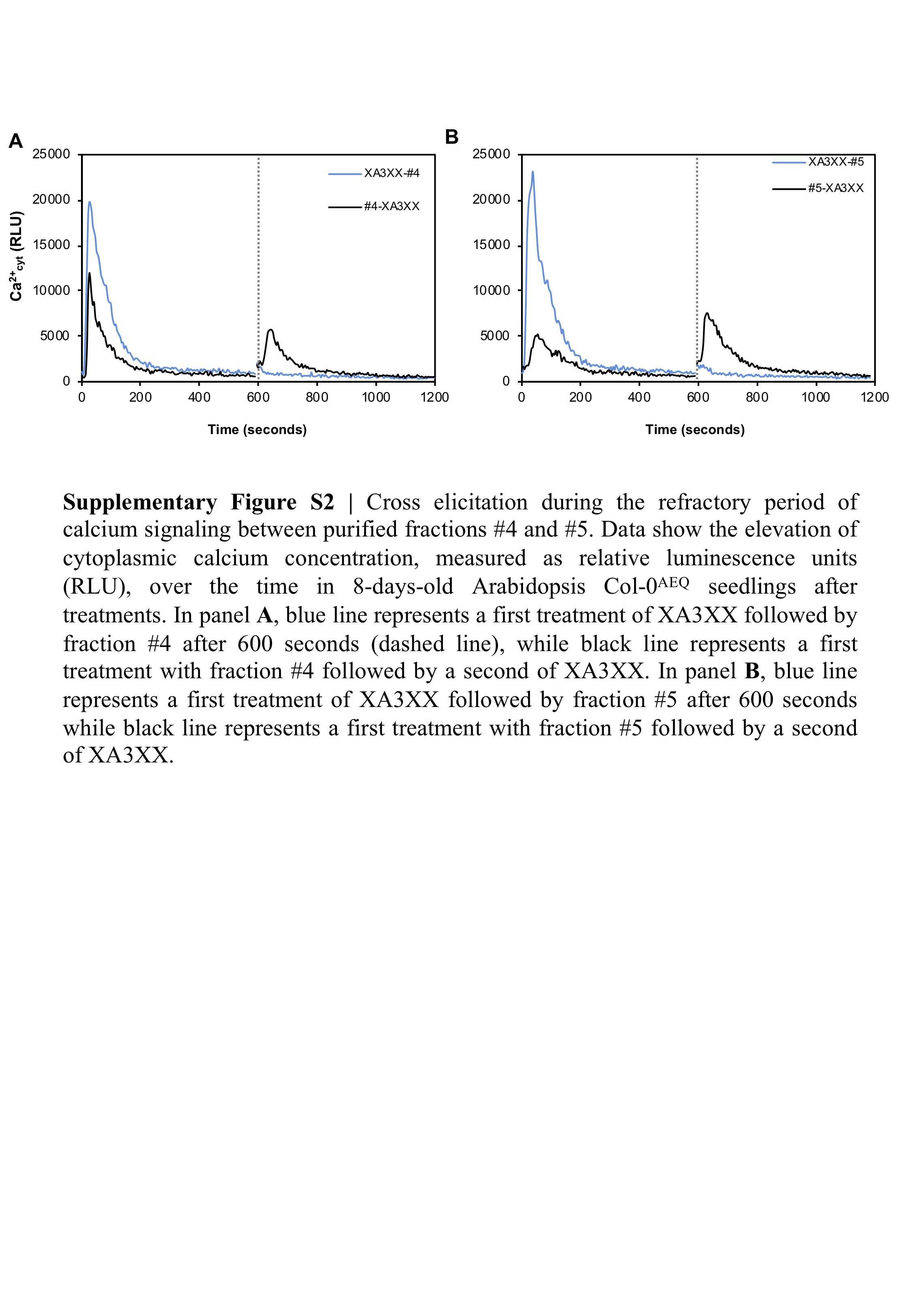

Supplement: Supplementary file 6 [file Image_2.tiff]

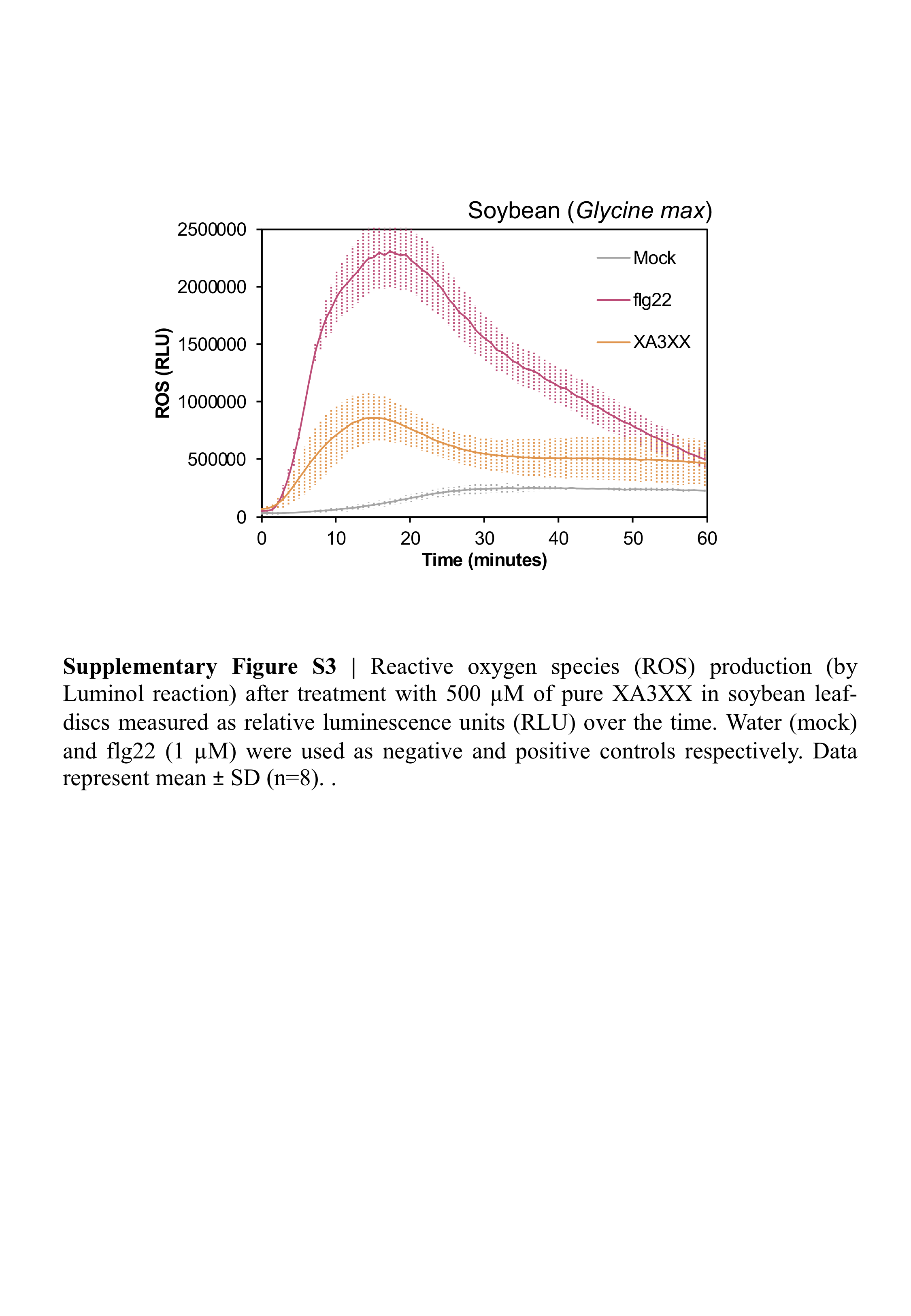

Supplement: Supplementary file 7 [file Image_3.tiff]
